# The exception that proves the rule: Virulence gene expression at the onset of *Plasmodium falciparum* blood stage infections

**DOI:** 10.1371/journal.ppat.1011468

**Published:** 2023-06-29

**Authors:** Jan Stephan Wichers-Misterek, Ralf Krumkamp, Jana Held, Heidrun von Thien, Irene Wittmann, Yannick Daniel Höppner, Julia M. Ruge, Kara Moser, Antoine Dara, Jan Strauss, Meral Esen, Rolf Fendel, Zita Sulyok, Myriam D. Jeninga, Peter G. Kremsner, B. Kim Lee Sim, Stephen L. Hoffman, Michael F. Duffy, Thomas D. Otto, Tim-Wolf Gilberger, Joana C. Silva, Benjamin Mordmüller, Michaela Petter, Anna Bachmann

**Affiliations:** 1 Bernhard Nocht Institute for Tropical Medicine, Hamburg, Germany; 2 Centre for Structural Systems Biology, Hamburg, Germany, Hamburg, Germany; 3 Biology Department, University of Hamburg, Hamburg, Germany; 4 German Center for Infection Research (DZIF), partner site Hamburg-Borstel-Lübeck-Riems, Hamburg/Borstel/Lübeck/Riems, Germany; 5 Institute of Tropical Medicine, University of Tübingen, Tübingen, Germany; 6 German Center for Infection Research (DZIF), partner site Tübingen, Tübingen, Germany; 7 Institute of Microbiology, University Hospital Erlangen, Friedrich-Alexander-University Erlangen-Nürnberg, Erlangen, Germany; 8 Institute for Genome Sciences, University of Maryland, School of Medicine, Baltimore, Maryland, United States of America; 9 Cluster of Excellence: EXC 2124: Controlling Microbes to Fight Infection, Tübingen, Germany; 10 Centre de Recherches Médicales de Lambaréné, Lambaréné, Gabon; 11 Sanaria Inc., Rockville, Maryland, United States of America; 12 Department of Microbiology and Immunology, Peter Doherty Institute for Infection and Immunity, The University of Melbourne, Melbourne, Victoria, Australia; 13 School of Infection & Immunity, University of Glasgow, Glasgow, United Kingdom; 14 Department of Microbiology and Immunology, University of Maryland, School of Medicine, Baltimore, Maryland, United States of America; 15 Global Health and Tropical Medicine, GHTM, Instituto de Higiene e Medicina Tropical, IHMT, Universidade NOVA de Lisboa, UNL, Lisboa, Portugal; Hebrew University, ISRAEL

## Abstract

Controlled human malaria infections (CHMI) are a valuable tool to study parasite gene expression *in vivo* under defined conditions. In previous studies, virulence gene expression was analyzed in samples from volunteers infected with the *Plasmodium falciparum* (Pf) NF54 isolate, which is of African origin. Here, we provide an in-depth investigation of parasite virulence gene expression in malaria-naïve European volunteers undergoing CHMI with the genetically distinct Pf 7G8 clone, originating in Brazil. Differential expression of *var* genes, encoding major virulence factors of Pf, PfEMP1s, was assessed in *ex vivo* parasite samples as well as in parasites from the *in vitro* cell bank culture that was used to generate the sporozoites (SPZ) for CHMI (Sanaria PfSPZ Challenge (7G8)). We report broad activation of mainly B-type subtelomeric located *var* genes at the onset of a 7G8 blood stage infection in naïve volunteers, mirroring the NF54 expression study and suggesting that the expression of virulence-associated genes is generally reset during transmission from the mosquito to the human host. However, in 7G8 parasites, we additionally detected a continuously expressed single C-type variant, Pf7G8_040025600, that was most highly expressed in both pre-mosquito cell bank and volunteer samples, suggesting that 7G8, unlike NF54, maintains expression of some previously expressed *var* variants during transmission. This suggests that in a new host, the parasite may preferentially express the variants that previously allowed successful infection and transmission.

**Trial registration:** ClinicalTrials.gov - NCT02704533; 2018-004523-36

## Author summary

Attachment of *Plasmodium falciparum*-infected erythrocytes to vascular endothelium via PfEMP1 is an important determinant of virulence and malaria severity, but these proteins also represent targets of the immune system and are therefore subject to antigenic variation by switching the expressed *var* gene. How parasites establish infection in a new host of unknown immunological history is poorly understood. Analyses of PfEMP1 expression during the course of controlled human malaria infections (CHMI) have previously focused on the parasite strain NF54 of African origin. In this study, we explored samples from volunteers infected with the South American parasite line 7G8 and confirm broad expression of subtelomeric *var* genes at the onset of blood stage infections, as initially discovered with NF54 infections. In addition, we expose the dominant expression of a single centromeric *var* gene before and after transmission as an alternative strategy of 7G8 parasites. Consistent with epigenetic retention of histone modifications during passage through mosquitoes, we provide evidence that the chromatin profile of *var* genes is maintained during sexual differentiation. This suggest that the PfEMP1 variant expressed in the previous malaria patient might also contribute to establishing an infection in the next human host.

## Introduction

Malaria caused by *Plasmodium falciparum* (Pf) remains one of the most serious global health problems, especially among children under the age of five. The clinical symptoms of malaria are exclusively associated with the erythrocytic stage of the parasite lifecycle, and virulence has been linked to the variant surface antigen Pf erythrocyte membrane protein 1 (PfEMP1) [1]. This immunodominant surface antigen is encoded by approximately 60 different *var* genes that differ in the composition of their adhesive extracellular protein domains, chromosomal location, and transcriptional orientation [2–4]. Group B *var* genes include the most telomeric genes on most of the 14 Pf chromosomes adjacent to group A *var* genes. A few group B *var* genes are also present in central chromosomal clusters along with group C *var* genes. The proteins encoded by group A are longer and more complex in domain composition than those of the other *var* gene groups [5]. Group A and B *var* genes containing an EPCR-binding CIDRα1 domain are more frequently associated with severe disease and complications in paediatric infections [6–11]. Group C *var* genes have been associated with mild malaria [12], and a single, highly conserved, subtelomeric, group E *var* gene encodes the VAR2CSA protein causing placental sequestration and placental malaria [13]. A few other variants (*var1*, *var3*) are also conserved in most Pf isolates, but their biological function is still unknown. Although the total number of *var* genes varies among Pf isolates, the proportion within each subgroup is relatively constant [5]. Recently, comparison of 15 genomes from geographically dispersed Pf isolates revealed that the highly polymorphic variable gene families exhibit little sequence homology, some copy number variation, but considerable consistency in their genomic organisation such as the orientation of the most telomeric *var* gene, positional conservation, and a fairly consistent number of *var* genes in internal clusters with similar orientation [14].

*Var* gene expression is monoallelic, i.e. each parasite expresses only a single variant at a given time [15]. Many factors have been found to contribute to the regulation of *var* gene expression, e.g., cis-acting elements such as the *var* promoter and intron [16,17], trans-factors [18,19], higher order chromatin structures [20,21], and epigenetic marks [22–26]. The majority of *var* genes is repressed in a heterochromatin environment characterized by the histone modification histone 3 lysine 9 trimethylation (H3K9me3), which is bound by heterochromatin protein 1 (HP1) [27–29]. The single active *var* gene is largely free of H3K9me3 and instead assumes a euchromatic structure with promoter enrichment of the histone variants H2A.Z and H2B.Z and histone acetylations including H3K9ac and H3K27ac [22,24,25,30]. Nevertheless, a comprehensive mechanistic understanding of how the mutually exclusive regulation of *var* expression occurs does not exist. Previous experiments examining switch rates in clonal cell lines showed that subtelomeric *var* genes have higher switch rates than central *var* genes [31,32], with A-type *var* genes rarely activated in *in vitro* cultures and so are not detected without selection pressure [33,34]. Mathematical modeling supports the idea of a non-random, highly structured switch pathway in which an originally dominant transcript switches either to a new dominant transcript or back to the previous one via a set of switch intermediates [35]. The *var2csa* gene of group E was previously proposed to be one such intermediate [36,37], and Zhang *et al*. have recently provided novel evidence for such a transcriptional network coordinated by *var2csa* [38]. Therefore, switching of *var* gene expression is determined by intrinsic activation/deactivation rates of *var* genes, suggesting that the frequency of antigenic variation is a balanced process between hierarchical *var* switches and selective forces in the host, such as host genetics and immunity.

Controlled human malaria infections (CHMI) provide a tailored environment to study parasite *var* gene expression *in vivo* under defined conditions, e.g., variables such as host immunity and infection time (i.e., the number of replication cycles of the parasite) can be monitored or even controlled. Previous results with the Pf isolate NF54 indicate that at some point during host-to-host transmission, the *var* expression profile is reset, allowing a phenotypically diverse population of parasites predominantly expressing different subtelomeric B-type *var* genes to enter the blood [39,40]. These results are consistent with observations in a murine malaria model, in which mosquito transmission of serially blood-passaged parasites resulted in broad activation of subtelomeric genes encoding variant surface antigens [41]. How this reset is achieved at the molecular level is currently also unknown.

In the past, the NF54 isolate was commonly used for CHMI, but recently other strains have become available for heterologous CHMI: 7G8 from Brazil, NF166.C8 from Guinea, and NF135.C10 from Cambodia. All of these strains have been shown to be representative of their geographic origin and to differ in their genome structure, sequence, and immunogenic potential [42,43]. The NF54 isolate was isolated from a Dutch patient who lived near Schiphol Airport, Amsterdam, and had never left the Netherlands. The infected mosquito responsible for this airport malaria case was probably imported from Africa [44]. In contrast, 7G8 was cloned from the IMTM22 isolate in 1984 and selected for its ability to produce microgametes, exflagellate, and infect *Anopheles freeborni* resulting in oocysts and sporozoites [45].

In the present study we show the first *in vivo* data on *var* gene expression from CHMI with malaria-naïve volunteers infected with the Pf clone 7G8 and provide new insights into the *var* gene expression pattern that Pf uses to establish a blood stage infection after transmission from one human host to another.

## Results

### The 7G8 *var* gene repertoire and the domain composition of the encoded PfEMP1 variants

The 7G8 *var* genome was extracted from previous whole-genome sequencing analyses, which are deposited on PlasmoDB Release 58 [14,42,46]. A total of 41 full-length putative *var* genes and three *var* genes with open reading frames corresponding to *var* exon 1 and exon 2 with a premature stop codon resulting in a truncated ATS domain, and therefore annotated as pseudogene on PlasmoDB, were identified, as well as 24 shorter *var* pseudogenes (S1 Table). In this study, we included all 41 full-length *var* genes, the three exon 2 truncated *var* pseudogenes, and to account for the presence of multiple *var* fragments in the 7G8 genome, also a single short C-type *var* fragment (Pf7G8_120024200) (S1 Table) (total n = 45).

**Fig 1:**
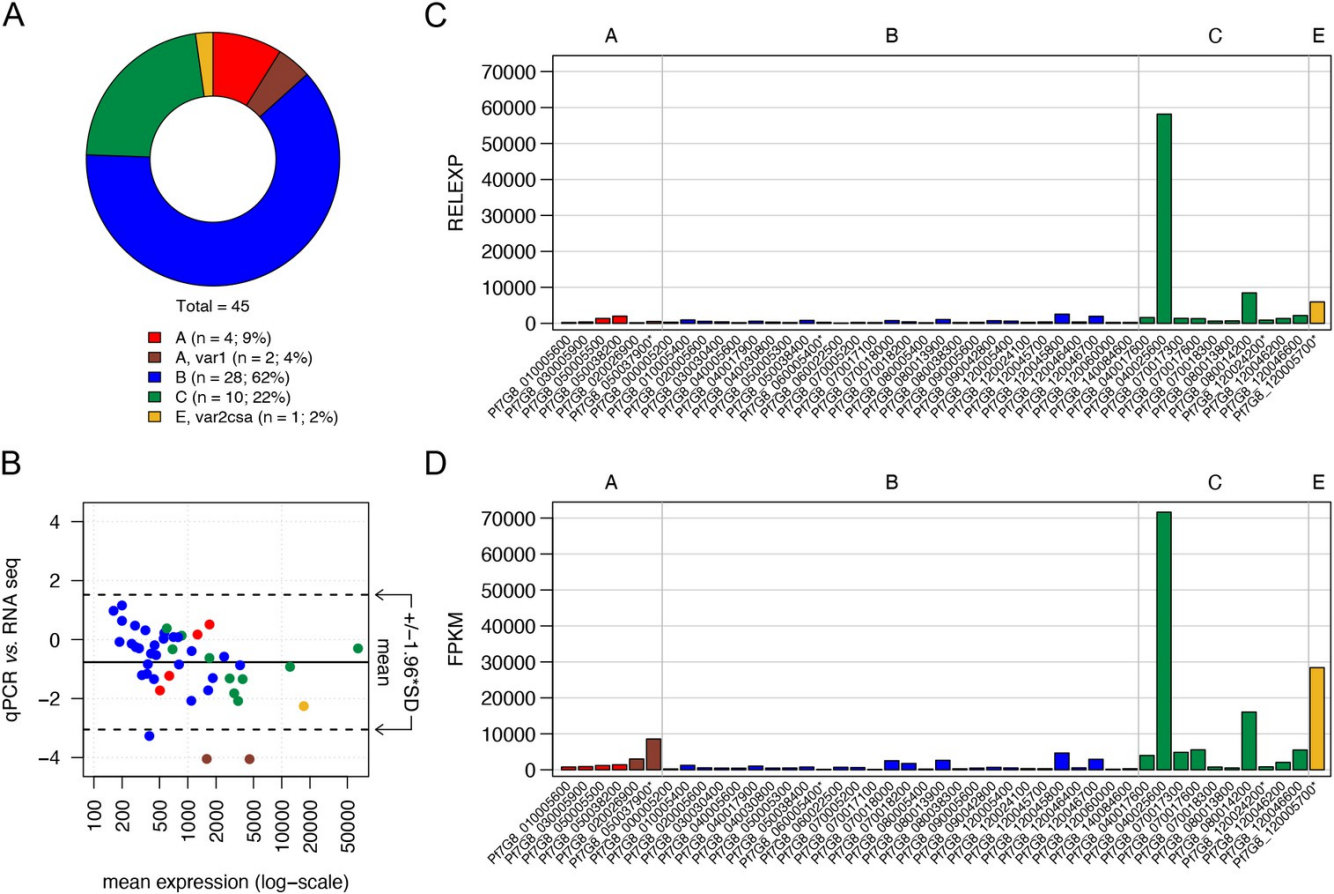
Validation of 7G8-specific qPCR covering the full *var* gene repertoire. (**A**) The genomic proportion of each *var* gene group in 7G8 parasites, including the three pseudogenes *var1-3D7* (Pf7G8_050037900), *var2csa* (Pf7G8_120005700), and the B-type Pf7G8_060005400 as well as the short C-type *var* fragment Pf7G8_120024200 (total n = 45). (**B**) RNA-seq and qPCR expression data shown in a Bland-Altman-Plot where the mean expression of each gene is shown on the X-axis and the ratio between RNA-seq and qPCR values on the y-axis. The mean of all ratios and the confidence interval (CI) of 95% are indicated by lines. Outliers are the two *var1* genes (Pf7G8_020026900, Pf7G8_050037900) and a B-type gene (Pf7G8_060022500), which show higher expression in RNA-seq. (**C, D**) The RNA sample (cell bank parasites aliquot B, *in vitro* generation 13) analyzed via qPCR (**C**) or RNA-seq (**D**) shows a nearly identical expression pattern by both analysis methods. qPCR data show gene expression of each *var* gene relative to the normalizer *arginyl-tRNA synthetase*, RNA-seq data are presented in FPKM (Fragments Per Kilobase of transcripts per Million mapped reads) values. *Var* gene names are listed on the x-axis, *var* group affiliations are indicated in red (group A), dark red (group A, subfamily *var1*), blue (group B), green (group C), yellow (group E, *var2csa*). Annotated pseudogenes are marked with an asterisk.

The Pf clone 7G8 encodes six group A *var* genes, including two *var1* genes (IT- and 3D7-type [47]), 28 group B *var* genes, ten group C *var* genes (including the short Pf7G8_120024200 pseudogene) and one group E *var2csa* gene (S1 Table and Fig 1A) [14,42]. The three pseudogenes with premature stop within the ATS domain include *var1*-3D7, group E *var2csa*, and B-type Pf7G8_060005400 (S1 Table). Genes of the conserved *var3* subfamily are absent. Two *var* A genes encode CIDRα1 domains that presumably can bind EPCR, and the other two A-type variants have an N-terminal head structure with CIDRδ/γ domains of unknown binding capacity. All remaining B- and C-type *var* genes encode PfEMP1 with CIDRα2–6 domains responsible for binding the host’s CD36 receptor. In total, 24 genes of type A, B or E are located in subtelomeric regions (24/44 = 54.6%), although it is unclear on which chromosome the *var* gene Pf7G8_000005200 is located.

### *In vitro var* transcript profiles of cell bank 7G8 parasites at ring stage prior to mosquito passage

Based on the newly assembled and annotated 7G8 *var* gene set, we designed and validated gene-specific primer pairs for each *var* gene (S2 Table). Subsequent qPCR and RNA-seq analysis was performed side-by-side using RNA from ring-stage parasites prior to mosquito infection (Sanaria cell bank, aliquot B, generation 13). The Bland-Altman-Plot of the expression values shows high similarity between the two data sets with only three outliers, Pf7G8_020026900 (A, *var1-IT*), Pf7G8_050037900 (A, *var1-3D7*) and Pf7G8_060022500 (B-type), all three expressed at a low level (Fig 1B–1D).

**Fig 2:**
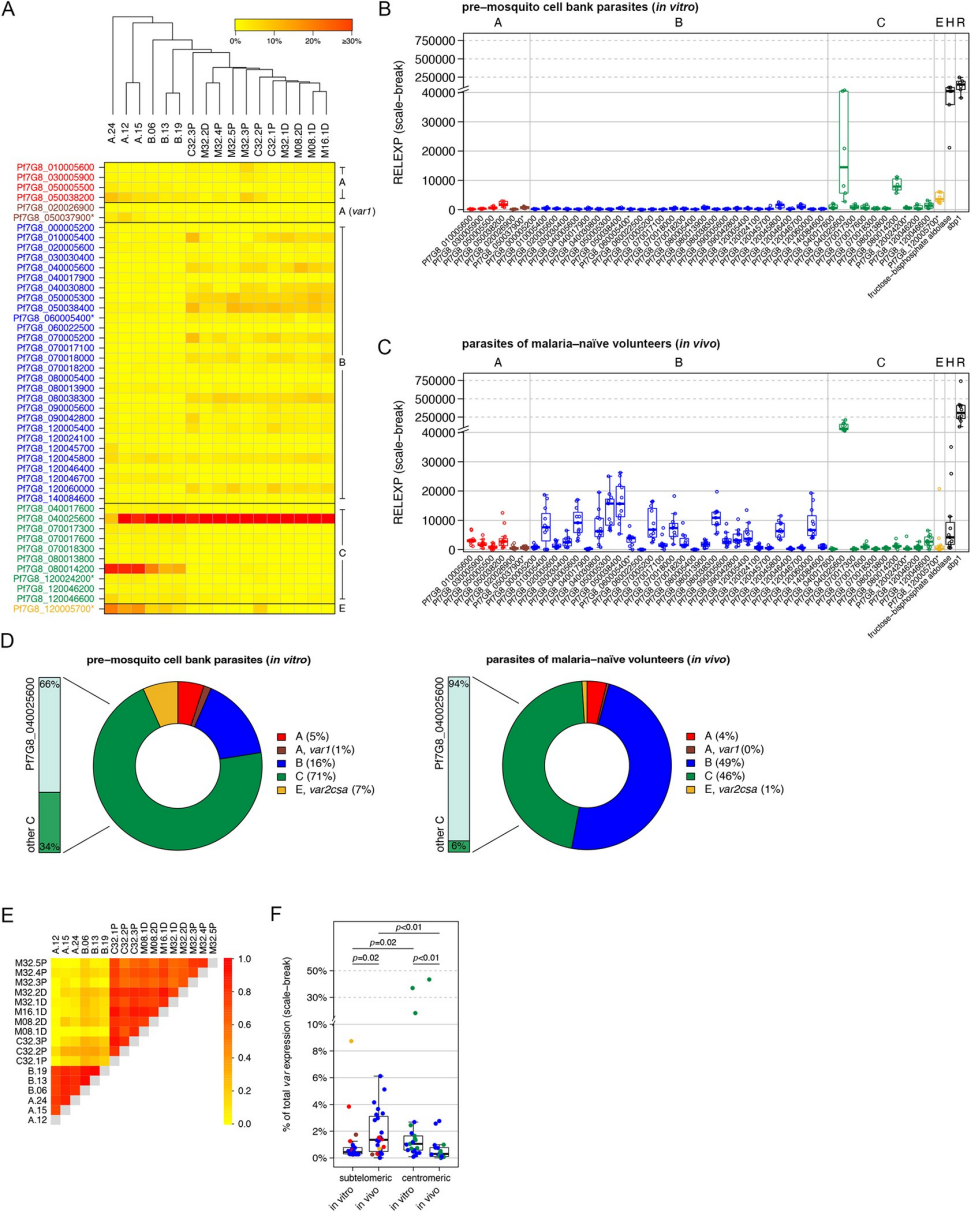
*Var* transcript profiles of pre-mosquito 7G8 cell bank parasites and of 7G8 parasites recovered from infected volunteers on the first day of detectable parasitemia. (**A**) Heat map showing individual *var* expression profiles for eleven volunteer samples (M08.1D, M08.2D, M16.1D, M32.1D, M32.2D, M32.3P, M32.4P, M32.5P, C32.1P, C32.2P and C32.3P) taken immediately before the start of treatment and six pre-mosquito cell bank parasite samples (aliquot A: *in vitro* generations 12, 15 and 24 post-thaw; aliquot B: *in vitro* generations 6, 13 and 19 post-thaw). To correct for individual differences in the total *var* expression levels, the expression for each *var* gene was normalized against total *var* expression in each sample. Hierarchical cluster analysis confirmed that pre-mosquito cell bank parasite samples differed from *in vivo* volunteer samples. (**B**, **C**) Gene expression of each *var* gene and controls relative to the normalizer *arginyl-tRNA synthetase* expression is shown in scatter plots for the pre-mosquito cell bank parasite line (**B**) and parasites obtained from the volunteers (**C**). Each point represents a value observed for the pre-mosquito samples taken from two independently thawed parasite stocks after 12, 15 and 24 (aliquot A) or 6, 13 and 19 parasite generations (aliquot B) and for eleven volunteer samples at day 11–13 after sporozoite inoculation. (**D**) Proportion of *var* gene expression by group for pre-mosquito cell bank parasites and parasites isolated from volunteers. (**E**) Heat map of pairwise Pearson correlation coefficients (PCC) between expression profiles illustrates the positive correlation between samples. (**F**) Comparison of the expression levels between subtelomeric and centromeric *var* gene variants in pre-mosquito cell bank parasites (‘*in vitro*’, aliquot A and B, n = 6) and parasites isolated from volunteers (‘*in vivo*’, n = 11). Each dot represents the mean proportion (%) of total *var* expression for each *var* gene variant located in either the subtelomeric or centromeric region of the 7G8 genome. The boxes represent medians with interquartile range (IQR); the whiskers depict minimum and maximum values (range) with outliers located outside the whiskers. Statistical analyses were performed using the Mann Whitney U test. Group affiliation of *var* genes is indicated by the color code with A-type *var* genes in red, the subfamily *var1* in dark red, B-type genes in blue, group C genes are colored in green and the *var2csa* gene (group E) is shown in yellow. Control genes are shown in black. Annotated pseudogenes are marked with an asterisk. H: housekeeping gene, *fructose-bisphosphate aldolase*; R: ring control, *skeleton-binding protein 1* (*sbp1*).

In addition, we performed comparative qPCR analysis of two cell bank aliquots A and B to probe into putative variations within the parasite population used for sporozoite production. We showed that the *var* gene expression pattern in both aliquots is dominated by a centromeric group C *var* gene variant, Pf7G8_040025600, which accounts for 30.3% (aliquot A, generation +12 after thawing) and 43.3% (aliquot B, generation +6 after thawing) of the total *var* gene transcripts, followed by a second centromeric type C *var* gene, Pf7G8_080014200 (aliquot A: 25.2%; aliquot B: 14.9%), and the group E *var2csa* gene, Pf7G8_120005700 (aliquot A: 10.8%; aliquot B: 4.5%) (Fig 2A and 2B and S3 Table). This specific expression pattern was revalidated in both cell bank aliquots after additional *in vitro* proliferation cycles (aliquot A: +15, +24; aliquot B: +13, +19). It was found that the overall pattern of *var* gene expression among each aliquot remained reasonably similar over time, though Pf7G8_040025600 expression declined in aliquot A at the last time point and gene expression of variants such as *var2csa* or Pf7G8_080014200 also varied slightly over time (Fig 2A and S3 Table).

### *In vivo var* transcript profiles in malaria-naïve volunteers infected with 7G8 sporozoites

Next, we analyzed the *var* gene expression profiles in samples from 7G8-infected volunteers from two different clinical trials, MAVACHE [48] and CVac-Tü3 [49] (S1 Fig). Our analysis of *var* gene expression profiles in parasites from eleven individual malaria-naïve volunteers (Table 1) showed that transcripts of all 45 *var* gene variants included in our study were detectable after 2–3 replication cycles of 7G8 blood stages (median: 11 days post infection, IQR: 11–12), similar to the expression pattern previously observed in NF54 [40]. Interestingly, all volunteer samples showed a very similar *var* gene expression pattern during this early blood stage infection (Fig 2A, 2C, 2D and 2E) dominated by the same single centromeric group C *var* gene variant, Pf7G8_040025600, accounting for a median 41.0% (IQR: 37.7–49.4) of total *var* gene expression (S3 Table). In addition, a broad induction of subtelomeric *var* genes, mainly group B, was observed (Fig 2A, 2C and 2D). Besides the dominant Pf7G8_040025600, the next ten most highly expressed *var* variants, each accounting for more than 2% of total *var* gene expression, are classified as B-type, and nine of them are located in subtelomeric regions (S2 Fig and S3 Table). Hierarchical cluster analysis and pairwise Pearson correlation coefficients of samples from pre-mosquito cell bank parasites and malaria-naïve infected volunteers showed clearly distinct expression profiles except for the dominant C-type Pf7G8_040025600 highly expressed in both (Fig 2A and 2E). Consistent with this, a comparison of expression levels between subtelomeric and centromeric located *var* genes in parasites from malaria-naïve volunteers and pre-mosquito cell bank parasites revealed significant differences between gene subsets. In general, significantly higher expression of subtelomerically located genes compared to centromeric genes was observed *in vivo*, whereas the reverse was observed *in vitro* (Fig 2F).

**Table 1:** Overview of volunteer characteristics infected with PfSPZ Challenge 7G8 and parasite counts determined either by thick blood smear (TBS) or qPCR at the day of treatment/sampling.

Volunteer ID	Sex	Year of birth	Trial phase/Immunization	No. of 7G8 sporozoites	Day of treatment/sampling	Parasites/μL (TBS)	Parasites/mL (qPCR)
M08.1D	m	1976	Dose optimization	800	13	11	8,216
M08.2D	m	1988	Dose optimization	800	12	7	717
M16.1D	m	1992	Dose optimization	1,600	12	30	8,593
M32.1D	m	1989	Dose optimization	3,200	11	0*	3,671
M32.2D	m	1993	Dose optimization	3,200	12	89	3,956
M32.3P	f	1992	Regimen verification (placebo)	3,200	10	3	3,560
M32.4P	f	1994	Regimen verification (placebo)	3,200	11	0*	1,757
M32.5P	m	1991	Regimen verification (placebo)	3,200	11	0*	3,666
C32.1P	f	1992	Regimen verification (placebo)	3,200	11	4*	15,559
C32.2P	m	1993	Regimen verification (placebo)	3,200	10	0*	8,955
C32.3P	m	1997	Regimen verification (placebo)	3,200	11	0	11,555

* Below lower limit of detection of TBS

### Characterization of dominant C-type *var* gene expressing parasites

**Fig 3:**
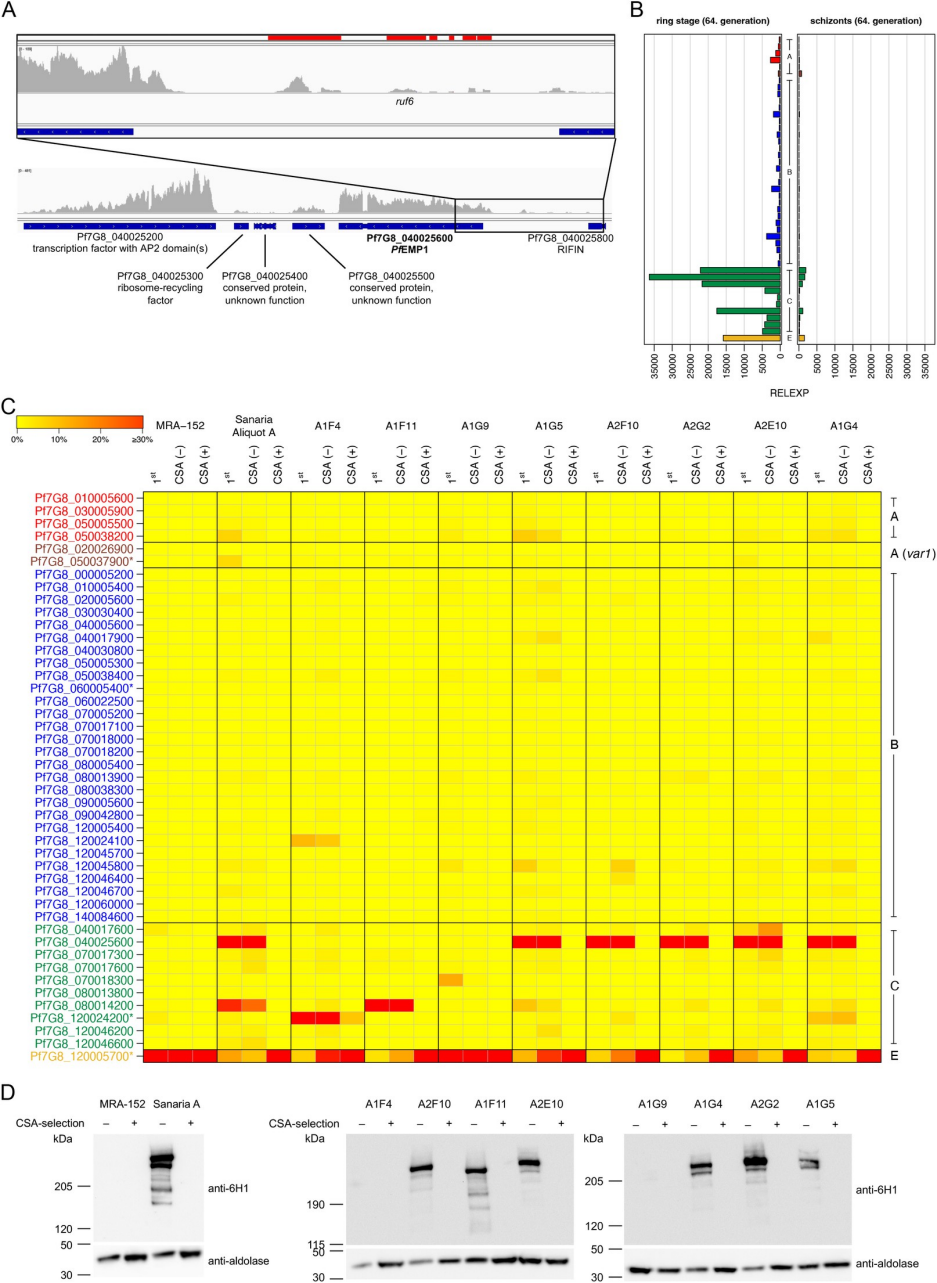
Characterization of parasites expressing Pf7G8_040025600. (**A**) Mapping of RNA-seq reads to the locus around the Pf7G8_040025600 gene in cell bank parasite aliquot B (13. *in vitro* generations after thawing). (**B**) Correct down-regulation of *var* gene expression as the parasite matures from the ring to the schizont stage (cell bank aliquot A). *Var* gene groups are indicated in the middle of the diagrams. (**C**) Selection of bulk cultures and newly generated subclones on CSA. Parasites obtained from BEI resources (MRA-152) and pre-mosquito cell bank parasites aliquot A (Sanaria A) and eight selected subclones derived from pre-mosquito cell bank aliquot A were enriched for CSA-binding. For each cell line, v*ar* gene expression is shown at three time points: (i) at the earliest time point after thawing or appearance after cloning (1^st^), (ii) before (CSA -), and (iii) after (CSA +) CSA selection. As expected from the *var* expression pattern, MRA-152 and the subclone A1G9 bound immediately to CSA due to the high expression of *var2csa* in the population. After three rounds of selection, pre-mosquito cell bank parasites and all other subclones were also enriched for CSA binding and *var2csa* expression. (**D**) Western blots of pre-selected and CSA-selected parasites using an anti-ATS antibody (6HI) and anti-aldolase as loading control. The truncation of the ATS sequence of 7G8-VAR2CSA hinders recognition of the protein by anti-ATS 6HI, explaining why PfEMP1 signal is lost in all parasite lines after CSA selection. Similarly, MRA-152, A1G9 and A1F4 show no signal due to expression of *var2csa* (MRA-152, A1G9) or the truncated pseudogene Pf7G8_120024200 (A1F4) before selection. *Var* gene names are indicated and annotated pseudogenes are marked with an asterisk, *var* gene groups are colored according to the following scheme: A in red, A-*var1* in dark red, B in blue, C in green and E (*var2csa*) in yellow.

All 11 samples from 7G8-infected volunteers analyzed in this study had the same *var* expression signature: The C-type *var* gene Pf7G8_040025600 was the dominant transcript that did not appear to be reset during transmission. This might suggest that this gene is mis-regulated in Sanaria cell bank 7G8 parasites in a way that it is either i) permanently activated, or ii) escapes from the mutually exclusive epigenetic silencing machinery, or iii) not subject to resetting during transmission [39,40]. Pf7G8_040025600 is the only *var* gene present in a central cluster of variant surface antigen genes on chromosome 4, where most other Pf strains have multiple *var* genes [14], and which also includes a *ruf6* sequence, a *rif* gene, and a *var* pseudogene. Pf7G8_040025600 has a relatively short intron with a length of only 253 bp compared to *var* genes of the Laverania subgenus (~800–1,200 bp) and all 7G8 variants (median 861.5 bp, range 245–1,348 bp) (S2 Table) [50], but shortened repeat regions 1–3 could be identified. To probe into the first hypothesis, we re-evaluated the strand-specific RNA-seq transcriptome data from the pre-mosquito cell bank parasites (aliquot B) but found no apparent activation of the other genes near Pf7G8_040025600. Some smaller non-coding transcripts were expressed at low level from upstream of this locus, including a *ruf6* sequence known to positively regulate neighboring *var* genes [51] (Fig 3A). Intron-derived non-coding antisense transcripts could be observed at low levels (S3 Fig), consistent with their described function in activation of the cognate *var* gene [52].

Second, to ensure that Pf7G8_040025600 expression did not exhibit an aberrant expression profile during asexual blood stage development, we tightly synchronized 7G8 parasites from the cell bank and performed qPCR on ring and schizont stage parasites. The data showed that Pf7G8_040025600 expression, as well as expression of all other minor *var* transcripts, was downregulated upon parasite maturation, as expected: Schizonts showed a 22-fold reduction in relative expression compared to ring stage parasites (relative expression values of 36,357.1 in rings and 1,659.1 in schizonts) (Fig 3B).

Third, to test if Pf7G8_040025600 escaped mutually exclusive expression, we panned pre-mosquito cell bank parasites (aliquot A) on recombinant CSA, allowing selection of parasites expressing the CSA-binding PfEMP1 variant encoded by the *var2csa* gene [53]. In parallel, an aliquot of 7G8 parasites obtained from BEI Resources (MRA-152) was subjected to the same procedure as a control for CSA binding. Immediately prior to selection on CSA, the pre-mosquito cell bank parasites expressed Pf7G8_040025600 (48.7%), Pf7G8_080014200 (17.5%) and the *var2csa* Pf7G8_120005700 at a much lower level (7.0%). In 7G8 MRA-152 parasites, the *var2csa* gene Pf7G8_120005700 clearly dominated the expression pattern even without any selection on CSA (96.2%), which was also the case in the two other 7G8 lines deposited at BEI Resources by different providers (MRA-154: 97.9%; MRA-926: 74.6%) (S4 Fig). After three rounds of selection, both 7G8 lines expressed the *var2csa* gene Pf7G8_120005700 almost exclusively (cell bank aliquot A: 99.2%; MRA-152: 98.9%) (Fig 3C and 3D and S4 Table). The conserved *var2csa* gene is known to encode the ligand for CSA [53], although it was annotated as a protein-coding pseudogene in 7G8 on PlasmoDB Release 58 due to a premature stop codon at position 8,622 bp resulting in a truncated ATS sequence lacking the terminal 220 amino acids (S1 Table). However, in agreement with recent reports, our data suggest that the 7G8 *var2csa* gene encodes a fully functional protein that is exported to the host cell surface and can bind CSA *in vitro* [54,55].

Fourth, another explanation for the high expression of the C-type gene Pf7G8_040025600 in pre-mosquito cell bank parasites as well as in parasites infecting the volunteers could be a very strong promoter or preference of the 7G8 strain used for CHMI to switch to Pf7G8_040025600 expression. This was investigated by cultivating the CSA-selected cell bank parasite aliquot A for an additional 100 parasite replication cycles. At every 10^th^ parasite generation, the *var* gene expression pattern was analyzed by qPCR, which was surprisingly stable over time with almost exclusive expression of the *var2csa* gene (S5 Fig and S4 Table). The expression of Pf7G8_040025600 was rather low, with a median relative expression value of 189.3 (IQR: 61.1–303.1) compared to the median *var2csa* expression value of 357,486.3 (IQR: 260,004.0–563,871.6). These data indicate that predominant or frequent switching to Pf7G8_040025600 expression in the 7G8 strain used is unlikely to be a major factor in upregulated expression of this gene in pre- and post-mosquito samples.

So far, all these data supported normal regulation of the Pf7G8_040025600 gene. However, because analyses at the parasite population level could miss individual variations in single parasite clones, we produced 13 clones from the cell bank aliquot A after 36 *in vitro* replication cycles by limiting dilution. The expression pattern of *var* genes was determined by qPCR, and eight clones were selected for further comparison: five of the clonal cell lines showed distinct expression of Pf7G8_040025600 (A1G4, A1G5, A2E10, A2F10, A2G2), while three cell lines dominantly expressing other *var* genes of type C (A1F4: Pf7G8_120024200, A1F11: Pf7G8_080014200) or E (A1G9: Pf7G8_120005700/*var2csa*) were chosen as controls (Fig 3C and 3D). These *var* expression patterns further suggest that the mechanism of mutually exclusive expression regulation in Pf7G8_040025600-expressing cell lines is still intact. Moreover, the successful selection of subclones for CSA binding revealed that *var* gene expression is not permanently fixed to this variant but can still switch to *var2csa*, which was confirmed at the RNA and protein levels (Fig 3C and 3D). The presence of all *var* genes on gDNA level in each subclone was verified by qPCR (S6 Fig).

To identify genomic alterations in 7G8 cell bank subclones possibly associated with persistent expression of Pf7G8_040025600 after transmission, the subclones A2E10, A2G2 (both expressing Pf7G8_040025600), and A1G9 (control) were subjected to whole genome sequencing. After stringent filtering, both Pf7G8_040025600-expressing clones had mutations of A to G at position 886709 on chromosome 11, which is located in the intron of the ERO1-encoding gene Pf7G8_110027600 (putative endoplasmic reticulum oxidoreductin), and at position 679487 on chromosome 12 within the coding region of gene Pf7G8_120022100 (putative sno-RNA-associated small subunit rRNA processing protein), reflecting a synonymous exchange. Clone A2G2 had two additional single nucleotide mutations on chromosome 10 at positions 1015567 (C->T, non-synonymous) and 1015545 (T->C, synonymous), both within Pf7G8_100029900, which encodes a conserved membrane protein of unknown function. Overall, whole genome sequencing revealed no non-synonymous changes between the subclones expressing the Pf7G8_040025600 gene and the *var2csa*-expressing control (S5 Table), making it unlikely that genetic differences between the two subpopulations cause reset failure.

**Fig 4:**
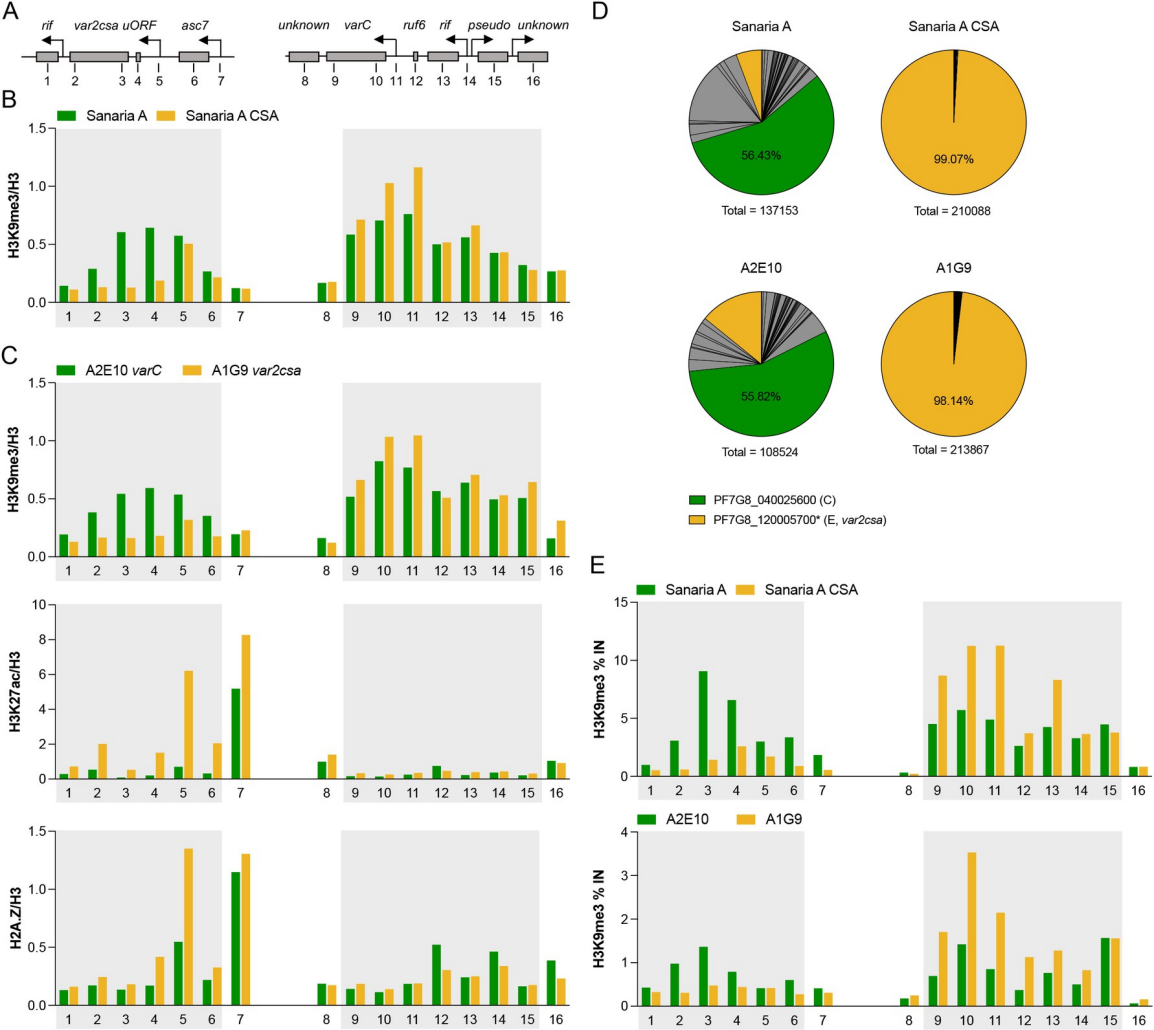
Epigenetic marks at the Pf7G8_040025600 and *var2csa* loci. (**A**) Genomic map of *var2csa* and Pf7G8_040025600 (‘*varC*’) loci with surrounding open reading frames (ORFs) and transcription start sites (indicated by arrows). The positions of the respective primer pairs used for ChIP-qPCR are indicated by numbers. (**B**) Chromatin immunoprecipitation with anti-H3K9me3 antibody and quantification of associated gDNA regions of *var2csa* (left panel) and Pf7G8_040025600 loci (right panel) by qPCR (ChIP-qPCR) in ring-stage Sanaria pre-mosquito cell bank parasites (aliquot A, ’*Sanaria A*’) and the same parasite line enriched for CSA-binding (’*Sanaria A CSA*’) (n = 1). ChIP data were normalized to ChIP input and H3 levels determined in parallel. (**C**) Levels of gDNA marked with the heterochromatin-associated H3K9me3, the activation mark H3K27ac, or the alternative histone variant H2A.Z associated with active gene expression were determined by ChIP-qPCR at different regions of the *var2csa* (left panels) and Pf7G8_040025600 (right panels) loci in subclones A2E10 (expressing C-type Pf7G8_040025600) and A1G9 (expressing E-type *var2csa*) (n = 1). (**D**) The corresponding expression profiles of *var* genes from RNA obtained in parallel with nuclei for ChIP experiments are shown as pie charts, with *var*2csa and Pf7G8_040025600 colored as indicated and all remaining *var* gene variants colored grey. Summarized total *var* gene expression is indicated for each parasite line. (**E**) ChIP-qPCR with anti-H3K9me3 antibody of gametocytes from Sanaria A and Sanaria A CSA (stage III) and from subclonal lines A2E10 and A1G1 (stage III/IV) at different regions of the *var2csa* (left panels) and Pf7G8_040025600 (right panels) loci. ChIP data are shown as % of input. The boundaries of the predicted heterochromatin regions are shaded in B, C and E.

Finally, to exclude epigenetic differences in gene regulation, we also performed ChIP-qPCR using ring stages from the cell bank aliquot A (bulk culture) and the same parasite line enriched for CSA-binding, as well as the cell bank subclones A2E10 (expressing Pf7G8_040025600) and A1G9 (*var2csa*-expressing control) (Fig 4 and S6 Table). Several epigenetic marks typically involved in *var* gene regulation were inspected, including the heterochromatin mark H3K9me3, which is associated with silent *var* genes [22], the euchromatic mark H3K27ac, which is typically enriched in active *var* promoters [30], and the histone variant H2A.Z, which occupies active *var* promoters as well as *var* introns regardless of expression status [24]. Both, the CSA-enriched cell bank culture and the A1G9 subclone showed the expected epigenetic profile at their expressed *var2csa*-loci (low H3K9me3, high H3K27ac/H2A.Z) and the silenced Pf7G8_040025600 locus (high H3K9me3, low H3K27ac/H2A.Z). However, this was less obvious in unselected cell bank parasites as well as the A2E10 subclone with dominant Pf7G8_040025600 expression. The Pf7G8_040025600 gene was clearly heterochromatic at a similar level as the *var2csa* gene, and was only marginally enriched for activation marks at the promoter (H2A.Z, H3K27ac) (Fig 4B and 4C). However, at least a slight reduction of H3K9me3 in Pf7G8_040025600 expressing relative to *var2csa* expressing cultures was consistently observed at the Pf7G8_040025600 gene, indicating that the locus may be partially unpacked from heterochromatin in part of the population. Parallel RNA analysis revealed that selection of parasites on CSA indeed resulted in a homogenous population with very high *var2csa* expression, whereas the unselected cell bank parasites and the A2E10 subclone showed a more diverse pattern of *var* expression with lower levels of total *var* expression and Pf7G8_040025600 in particular, which may possibly explain the differences in signal intensity of the epigenetic marks (Fig 4D).

### Maintenance of the heterochromatin profile of the ring-stage active *var* gene during gametocytogenesis

To probe into the epigenetic mechanism by which *var* gene expression might be maintained upon transmission, we performed ChIP-qPCR using stage III–IV gametocytes from the same parasite cultures we used for ring-stage ChIP-qPCR (cell bank aliquot A +/- CSA-enriched, cell bank subclones A2E10 and A1G9). Intriguingly, the H3K9me3 profile of the previously active *var* gene seems to be maintained during gametocytogenesis, albeit a drastic down-regulation of the corresponding *var* transcripts was observed (Figs 4E and S7). This was observed for the subtelomeric *var2csa* locus in CSA-selected cell bank aliquot A parasites and the subclonal line A1G9, as well as for the centromeric located Pf7G8_040025600 in unselected cell bank aliquot A parasites and the A2E10 subclone.

Overall, we demonstrated that expression of the C-type gene Pf7G8_040025600 (i) is a unique feature of parasites from the Sanaria cell bank, (ii) is correctly regulated across asexual blood stages, (iii) occurs in a mutually exclusive manner, and (iv) parasites are neither fixed to nor tend to preferentially activate expression of this variant after selection for *var2csa* expression. Moreover, parasites expressing this *var* variant share the genomic and epigenetic background with parasites expressing other *var* variants, which further rules out the presence of two distinct 7G8 parasite populations in the original cell bank aliquots, with one population behaving like NF54 and resetting *var* gene expression during mosquito passage, and the other population having lost its ability to reset *var* expression and being fixed to express Pf7G8_040025600. Therefore, our data rather support the second hypothesis, namely that Pf7G8_040025600 is not subject to resetting during transmission, suggesting that epigenetic resetting of *var* genes can be incomplete during passage through mosquitoes, possibly through the semiconservative retention of histone modifications at replicated *var* loci during meiosis. Consistent with this idea, we provide the first data showing that the heterochromatin profile of *var* genes in ring-stage parasites is maintained during gametocytogenesis.

## Discussion

After transmission, parasites are faced with a new environment defined by various parameters such as the metabolic state of the host, pre-existing immunity, drug pressure, pregnancy or other infections. One way to cope with these alterations might be to epigenetically reset genes upon transmission to create a diverse population of parasites expressing different *var* genes from which clones best adapted to the new environment can expand. This "bet-hedging" strategy, which is a viable alternative to directed transcriptional responses, is thought to be key to adaptations to general variations in the environment [56]. This has been shown, for example, for expression of *var* genes after liver release of NF54 parasites in CHMI of immunologically naïve individuals [39,40,57,58]. There, expression of *var* genes at the onset of infection shows a broad activation pattern of many group B subtelomeric genes and certain group A variants. This broad expression repertoire is reduced in NF54 parasites from infected volunteers with a higher degree of pre-existing immunity as soon as the parasites enter the blood phase. In these individuals, only a single or very few *var* gene variants are highly expressed, presumably encoding PfEMP1 which are not recognized by the host immune system [37]. Intriguingly, the *var* gene expression profile changed dramatically during mosquito passage of NF54 Pf parasites from the monoallelic *var2csa* expression in the initial culture used for gametocytogenesis and sporozoite production to the broad pattern of subtelomeric *var* gene transcription in the infected volunteers [40,58,59].

In this study, we investigated whether the observations using NF54 parasites also hold true in other Pf isolates, or whether parasite strain-specific differences in *var* gene expression patterns can be observed. This is of particular importance because current vaccination concepts using whole parasites–either radiation-attenuated sporozoites (PfSPZ Vaccine) or sporozoites co-administered with chemoprophylaxis (PfSPZ-CVac)–are based on the strain NF54. For the first time, we have obtained expression data for the entire *var* gene repertoire in another parasite strain, 7G8, which originates from a different geographic region and is genetically distinct from NF54 [42,43]. When 7G8 parasites were used for CHMI, a very similar pattern of *var* gene group B activation was observed in naïve volunteers. This suggests that Pf generally preferentially activates subtelomeric *var* genes after release from the liver, while central *var* genes tend to remain silent [40], allowing a phenotypically diverse population of parasites expressing different subtelomeric *var* genes to enter the blood from the liver. These results are consistent with observations in a mouse malaria model, where transmission of serially blood-passaged parasites by mosquitoes resulted in broad activation of subtelomeric genes encoding variant surface antigens [41]. How this switch occurs at the molecular level is currently unknown, but there is strong evidence that the regulation of *var* genes in Pf relies on epigenetic mechanisms [59–61]. In other systems with mutually exclusive expression, such as VSG expression in African trypanosomes and olfactory gene (OR) expression in mammals, there are intermediate developmental stages in which weakly expressed transcripts of multiple VSG or OR genes are detected that ultimately converge to high-level monoallelic expression of a single VSG/OR [62,63]. One might speculate that each Pf parasite undergoes the same procedure of probing the expression of multiple *var* genes before predominantly selecting a single subtelomeric B-type variant for mutually exclusive expression in the early blood phase, and this leads at the parasite population level to the broad B-type expression pattern that we observe for NF54 and also for 7G8 parasites. However, it is not known whether Pf employs a similar mechanism to "decide" which *var* variant to express at the onset of infection in the blood stage and when this decision is actually made. To investigate this question, single-cell data from different developmental stages of the parasite would be required, as in the studies on the regulation of VSG/OR genes. The broad activation pattern of many *var* genes seems to contradict the concept of safeguarding the antigenic repertoire. However, this strategy of releasing a parasite population from the liver that expresses PfEMP1 proteins with diverse adhesive properties would allow optimal and rapid adaptation to a new host environment shaped by selective forces such as host genetics and pre-existing immunity. At the same time, maintaining a repertoire of *var* gene variants silenced after liver release would prevent the parasites from exposing their entire antigenic repertoire too early. Differences in intrinsic switching rates of individual *var* genes would then lead to the expansion of parasite populations that express dominant PfEMP1 variants during the course of infection and are further selected by immunity, consistent with the fashion observed in chronic infections [64].

Interestingly, the dominant C-type *var* gene Pf7G8_040025600 in our study resists resetting during transmission. There are at least five possible explanations for the high expression of this variant in pre-mosquito parasites and *in vivo* during infection of volunteers: (i) the gene might have escaped mutually exclusive expression, (ii) Pf7G8_040025600 might be generally deregulated during asexual blood stage development, (iii) a subpopulation of parasites might have a mutation or carry epigenetic marks making this variant resistant to the resetting mechanism during mosquito passage, (iv) this *var* gene variant could have a very active promoter that allows high expression levels under different conditions, or (v) only parasites expressing *var2csa* or other *var* gene variants that act as switching hubs are able to reset their *var* gene expression during transmission. We tested most of these hypotheses and demonstrated that Pf7G8_040025600 expression is mutually exclusive with that of other *var* genes, and the gene is correctly repressed in schizonts, does not have a higher on switch rate than other *var* gene variants, and none of the few genetic differences observed between clonal Pf7G8_040025600- and *var2csa*-expressing parasites can explain the phenotype. Moreover, these parasites were still able to switch their *var* expression to a different variant, consistent with the successful generation of 7G8 sporozoites by Sanaria, suggesting that the switch from asexual replication to gametocytogenesis is also not hindered in these parasites. Our results from ChIP-qPCR also suggest that epigenetic regulation of Pf7G8_040025600 is not affected, as the gene is normally silenced by heterochromatin. The absence of detection of high levels of activating histone marks may be explained by the mixed *var* expression pattern in about half of the population, as it is known that in parasite populations with heterogeneous *var* gene expression, signals from ChIP-qPCR or -seq can be leveled out [58,65]. However, we found that the patterns of heterochromatin distribution at the previously active *var* locus in gametocytes resembled the profiles observed in ring stage parasites with active *var* expression. This observation is certainly not sufficient to explain the difference between NF54 and 7G8 in resetting their *var* gene expression, but provides a novel hint of the mechanisms by which *var* gene expression might be maintained during transmission.

Based on our data, we hypothesize that *var* gene expression may partly underlie epigenetic imprinting during transmission from one human host to another, which is thought to depend on the ability of each *var* gene locus to establish and maintain heterochromatin. Consistent with other studies showing that C-type *var* genes are turned on less frequently but can dominate expression patterns by slow silencing [31,66], C-type *var* genes may be less effective at re-establishing heterochromatin during transmission, in line with their location in central chromosomal regions with likely fewer Sir2A or B-dependent silencing nucleation sequences. In conjunction with the intrinsic promoter activity of each *var* gene variant, Pf could exhibit a loose activation hierarchy of subtelomeric *var* genes upon entry into the human blood phase. This strategy would allow the parasite population to explore a new host environment by expressing many different PfEMP1 variants as well as testing the previously successful variant in a different host, adding another layer to the parasite’s survival or adaptive strategies in humans. On the other hand, however, genetic restructuring of the centromeric *var* locus on chromosome 4 of 7G8 may have resulted in the loss or rearrangement of regulatory sequences that are also responsible for regulating the expression of Pf7G8_040025600. For example, truncation of intron region 2, sequences involved in nucleation of heterochromatin, or the entire locus might have been shifted closer to sequences such as *ruf6* that are involved in *var* activation [51]. Notably, intron region 2 has been previously identified in transient transfections with luciferase reporters as a critical region for repression of *var* gene expression through interaction with the cognate promoter [17,67], and this region 2 appears to be almost absent in the Pf7G8_040025660 intron. However, endogenous deletion of the *var2csa* intron did not support a role of the intron in transcriptional silencing, activation, or switching of *var2csa*, although in a single experiment easier reactivation of *var2csa* transcription by panning over CSA was observed in the intron-less parasites compared to wild type [68]. In addition, although we have shown that Sanaria cell bank 7G8 parasites do not switch from *var2csa* expression to Pf7G8_04025660 expression but on the contrary tend to downregulate Pf7G8_040025600 upon prolonged cultivation, it is also still possible, that the inherent promoter strength of Pf7G8_040025600 overwhelms the possibly altered heterochromatin nucleation signal at this locus, such that this gene is continuously activated during transmission despite the low accumulation of euchromatic markers. On top of all this, *var2csa* has also been shown to act as a switching intermediate [35–38], and it may also be possible that the resetting of *var* expression is coordinated only via intermediate *var2csa* expression, from which parasites can express *var* genes at the onset of the blood phase with probabilities based on intrinsic on-rates for each variant. It should be noted that truncation of VAR2CSA-ATS in 7G8 parasites may affect the efficiency of these processes. Potentially, both "*var* resetting phenotypes" during parasites transmission–the full reset of *var* gene expression observed in NF54 and the partial reset with maintenance of the previously active C-type *var* gene observed in 7G8 –co-exist in the parasite population. Further studies, such as CHMIs with NF54, 7G8, or additional strains expressing different *var* gene variants prior to transmission, or with parasites with genetically modified *var2csa* locus, are needed to evaluate these hypotheses.

Another interesting observation from CHMIs with adult volunteers infected with NF54 and 7G8 is the rather low proportion of parasites expressing A-type *var* genes, which have been associated with morbidity and mortality in children in malaria endemic areas. However, it is currently unclear whether adults are more likely to control these variants, which also results in a different, more multi-organ disease phenotype, or whether the sheer number of pediatric infections results in such a high number of severe and fatal cases. Available data from cross-sectional studies suggest that parasites from severely ill children express a higher proportion of A-types than parasites from adults with severe malaria, but to date a direct quantitative comparison of *var* gene expression of parasites in severely ill adults and children is lacking [10,69]. It may also be that most children are sicker than adults or that the parasites sequester in other sites in the child’s body, resulting in higher A-type levels. However, in contrast to CHMI with adults of endemic areas, A- or B/A-type expression associated with severity can be observed in CHMI with malaria-naive adults infected with NF54. The A- and B/A-types are only less abundant (13.5–23% of total *var* expression, depending on the study) than the B-types (about 75% of total *var* expression). For example, the A-type gene PF3D7_0400400 is the eighth most expressed gene in Bachmann *et al*. [40] and is also found at a relatively high level in other CHMI studies using NF54 [39,57,58]. If adhesion receptors select for A-types, these parasites could outcompete B-type-expressing parasites during the course of infection. In CHMI studies from malaria endemic regions, reduced expression of A-type was observed in volunteers with low immunity (‘non-controller’) and absence of expression in volunteers with high immunity (‘controller’), confirming that anti-A immunity indeed develops after only a few infections [37].

In summary, we demonstrated that (i) expression of subtelomeric B-type *var* genes is induced in 7G8 parasites at the onset of blood stage infection in malaria-naïve individuals, (ii) cell bank parasites used for PfSPZ production and isolated from volunteers exhibit an expression pattern dominated by a single C-type variant, Pf7G8_040025600, suggesting that this C-type variant underlies epigenetic memory during mosquito passage, which is further substantiated by maintenance of the heterochromatin profile in gametocytes. Our results from two genetically distant parasite backgrounds show that expression of virulence-associated genes in Pf is, at least partially, reset to express a broad repertoire of *var* genes, mostly of subtelomeric B-type, during transmission from mosquito to human host, but also provide evidence for an alternative strategy of the parasite in which infection in the next host is established by maintenance of expression of a previously successful PfEMP1 variant in part of the parasite population. This suggests that the PfEMP1 variant expressed in the previous malaria patient is an important factor that could determine the pathophysiology of the subsequent infection. In conclusion, the NF54 strain and its clone 3D7 appear to be an accurate reference for the entire species in terms of gene content and organization, as previously noted by Otto et al. [14], but there appear to be differences among Pf isolates in resetting *var* gene expression during transmission to establish infection in another human host [39,40].

## Methods

### Ethics statement

The ethics committee of the University Clinic and the Medical Faculty of the University of Tübingen approved both studies, MAVACHE (approval number: 023/2016AMG1) and CVac-Tü3 (approval number: 919/2018AMG1), of which samples were examines in this work, and the U.S. Food and Drug Administration Agency (FDA) provided regulatory oversight. The studies were conducted according to the principles of the Declaration of Helsinki in its 6th revision and the guidelines of the International Conference on Harmonization-Good Clinical Practice (ICH-GCP). For the MAVACHE study, the registration code at ClinicalTrials.gov is NCT02704533; the CVac-Tü3 trial is registered with the EU Clinical Trial Register under 2018-004523-36. All volunteers provided written informed consent, and understanding of the study and procedures was assessed with a quiz.

### CHMI trials and blood sampling

Samples were collected during different phases of the MAVACHE [48] and CVac-Tü3 trials [49]. Both trials were conducted at the Institute of Tropical Medicine in Tübingen, Germany, where healthy, malaria-naïve volunteers were infected with live, infectious, aseptic, purified, cryopreserved NF54 or 7G8 sporozoites (Sanaria PfSPZ Challenge (NF54) and Sanaria PfSPZ Challenge (7G8)) manufactured by Sanaria Inc., USA, 7G8 under license from Walter Reed Army Institute of Research. Participants were examined at least daily from day 6 after the start of CHMI and treatment was initiated when Pf parasitemia with asexual blood stages was detected by microscopy of the thick blood smear or parasitemia of more than 100 parasites per mL was detected along with two additional positive results, each at least 12 hours apart, by quantitative PCR. Clinical symptoms recorded during both studies (including subjects receiving PfSPZ vaccine or PfSPZ-CVac) were comparable to other CHMI studies with adverse events such as headache, fatigue, myalgia, pyrexia, and chills attributable to low parasitemia. On the day of treatment, up to 50 mL of blood was taken from all volunteers into sodium citrate tubes and processed by Ficoll gradient centrifugation followed by filtration of the washed red blood cell pellet through a Plasmodipur filter (EuroProxima).

The MAVACHE trial aimed to sequentially optimize the dose and schedule of PfSPZ Vaccine, verified by randomized, controlled, double-blind immunization and controlled human malaria infection in malaria-naïve, healthy adult volunteers in Germany. The dose optimization phase included a dose-finding phase to evaluate the safety, tolerability and infectivity of 7G8 PfSPZ in malaria-naïve, healthy adult volunteers. A total of nine volunteers received either 800 (n = 3), 1,600 (n = 3) or 3,200 (n = 3) 7G8 PfSPZ. Similar to NF54, 7G8 PfSPZ at a dose of 3,200 PfSPZ resulted in parasitemia in 3/3 of the volunteers, and 2/3 volunteers developed parasitemia after infection with 800 and 1,600 PfSPZ, respectively, all administered by direct venous inoculation. Parasite kinetics and clinical presentation are similar to CHMI with NF54 PfSPZ [70]. Samples from five volunteers are included in our study (800 PfSPZ: M08.1D, M08.2D; 1,600 PfSPZ: M16.1D; 3,200 PfSPZ: M32.1D, M32.2D) (Table 1 and S1 Fig). During the regimen verification phase, volunteers were either infected with PfSPZ Vaccine or received placebo, followed by a CHMI three weeks after the last immunization. The placebo group received normal saline instead of PfSPZ Vaccine, but also underwent subsequent CHMI. Samples from three volunteers infected with 7G8 PfSPZ (M32.3P, M32.4P, M32.5P) were included in our study (Table 1 and S1 Fig).

The CVac-Tü3 trial assessed the safety and protective efficacy of a simplified Pf sporozoite Chemoprophylaxis Vaccine (PfSPZ-CVac) regimen in healthy malaria-naïve adults in Germany [49]. In total, NF54 PfSPZ of PfSPZ-CVac was administered three times (day 0, 5 and 28) to volunteers receiving parallel chloroquine treatment (1.1 x 10^5^ PfSPZ each), and these volunteers underwent CHMI with 3,200 7G8 PfSPZ ten weeks after the last immunization. Samples from three placebo-infected volunteers (C32.1P, C32.2P, C32.3P) could be included into our study (Table 1 and S1 Fig).

In total, samples from eleven malaria-naive volunteers infected with 7G8 PfSPZ were included in this study.

### Parasite cell culture

7G8-MRA-152 (contributed by David Walliker), 7G8-MRA-154 (contributed by Dennis E. Kyle) and 7G8-MRA-926 (contributed by Karen Hayton and Tom Wellems) parasites were obtained through BEI Resources, NIAID, NIH. Two frozen vials (termed cell bank aliquots A and B) of 7G8 parasites from Sanaria’s working cell bank (lot: SAN03-021214 from 20. February 2014) were separately thawed and cultured in human O+ erythrocytes and in presence of 10% heat-inactivated human serum in parasite culture medium according to standard procedures [71]. To maintain synchronized parasites, 7G8 cultures were treated either with 5% sorbitol [72]. Tight synchronization was performed by percoll-enrichment of schizont [73] followed by sorbitol treatment after 4 hours of cultivation. From cell bank aliquot A ring stage parasites were collected at generations 12, 15 and 24 after thawing, from cell bank aliquot B at generations 6, 13 and 19.

Selection of *var2csa* expressing parasites was performed by panning on plastic dishes coated with 50 μg/mL bovine trachea CSA (*Sigma*), as described previously [74]. Subclones of Pf7G8 cell bank aliquot A were generated by limiting dilution cloning, as described previously [75].

Gametocytes were produced and maintained according to standard protocols. Briefly, sexual commitment was induced by culture in partially spent medium and inhibition of asexual stages with N-acetyl-D-glucosamine [76]. Chromatin and RNA were harvested on day 6 or 8 of gametocyte differentiation (stage III-IV).

### Western blot analysis

Trophozoite stage cultures were treated with 0.075% saponin in PBS to release hemoglobin from the erythrocytes. Parasites and membrane ghosts were pelleted by centrifugation, washed three times in PBS containing protease inhibitors (cOmplete EDTA free, Roche), and extracted in 2 x Laemmli buffer. The protein extracts were separated on 3–8% Tris-Acetate gels (Invitrogen) and transferred to nitrocellulose membranes (Millipore). The blots were probed with monoclonal mouse anti-ATS (6HI) antibody [77] or rabbit anti-*Plasmodium* aldolase antibody (abcam, ab207494) as loading control.

### gDNA purification for whole genome sequencing

For gDNA sequencing, 150 mL Pf cell culture with >10% parasitemia were harvested for the 7G8 cell bank aliquot A subclones A1G9, A2E10, and A2G2 and gDNA isolation was performed using the MasterPure Complete DNA purification kit (Lucigen) according to the manufacturer’s instructions “Cell samples” followed by “Complete Removal of RNA” with additional RNase I treatment. The gDNA samples were checked for degradation and RNA contamination on an agarose gel and quantified with the Qubit dsDNA BR Assay Kit (ThermoFischer).

### RNA purification and cDNA synthesis

Red blood cells were settled by centrifugation and completely lysed in 5 volumes of pre-warmed TRIzol (ThermoFisher). Samples were stored at -80°C until RNA purification. The RNeasy Mini kit with on-column DNase I treatment (Qiagen) was used for RNA purification. The absence of gDNA was checked for each sample using 50 ng RNA and the *skeleton-binding protein 1* (*sbp1*) primer set (S1 Table). cDNA synthesis was performed as previously described [37].

### Quantitative real-time PCR

The LightCycler 480 (Roche) was used for quantitative real-time PCR analysis using the provided LightCycler 480 software release 1.5.1.62 SP3 as previously described [37]. Briefly, cDNA template was mixed with QuantiTect SYBR Green PCR reagent (Qiagen) and 0.3 μM sense and antisense primer in a final volume of 10 μl. Reactions were incubated at 95°C for 15 min, then subjected to 40 cycles of 95°C for 15 s and 60°C for 1 min followed by a melting step (60–95°C). The specificity of each primer pair was confirmed by dissociation curve analysis after each qPCR run. Ct calculation was done using the fit points analysis method provided by the software. Expression of *arginyl-tRNA synthetase* (PF3D7_1218600) was used for normalization and Ct values obtained by analysis of 2.5 ng gDNA from Sanaria’s working cell bank parasites were used for calibration. Relative quantification of the 7G8 *var* repertoire by 2^-ΔΔCt^ analysis was performed using newly designed primer sets for 7G8 (S1 Table). Furthermore, primer pairs targeting the housekeeping gene *fructose-bisphosphate aldolase* as well as the ring stage control *sbp1* were included (S1 Table). Relative expression data (RELEXP) were corrected for amplification efficiency of each newly designed primer pair, which was determined by dilution of a single gDNA from 7G8 over 5–6 logs of concentration (S1 Table). For further characterization, the full 7G8 primer set was checked for cross-reactivity with NF54 gDNA [37], but only primers targeting the partial gene Pf7G8_120024200 produced a specific amplicon with NF54 gDNA. Only volunteer samples with Ct values below 30 for the housekeeping genes *fructose-bisphosphate aldolase* and *arginyl-tRNA synthetase* were included, as this has been shown to be a useful standard for analysis of *var* gene transcription in malaria patient and CHMI samples [37,40,78].

### RNA and gDNA sequencing

RNA from cell bank parasites (aliquot B, generation 13 after thawing) was purified and processed as previously described [79]. Briefly, absence of genomic DNA was checked, human globin mRNA was depleted, and RNA quantity and quality were assessed with an Bioanalyzer (RIN value 8.4). A 100–500 bp library was prepared using the NEBNext Ultra Directional RNA Library Prep Kit for Illumina including the amplification with the KAPA polymerase, and RNA sequencing on an Illumina HiSeq4000 was conducted by BGI Genomics Co., Hongkong. Approximately 12.6 million clean reads were obtained for pre-mosquito cell bank parasites, resulting in 6.3 million paired-end 100 bp reads.

Library construction of gDNA and paired-end 150 bp sequencing of approximately 350 bp fragments (range: 230–430 bp) on the DNBseq platform was done by BGI Genomics Co., Hongkong with coverage of approximately 130x (range: 129x–136x).

### RNA-seq data analysis

After successful quality control of RNA-seq reads with FastQC v0.11.8 (http://www.bioinformatics.babraham.ac.uk/projects/fastqc/) [80], the reads were aligned to the Pf 7G8 genome available from the PlasmoDB genome database release 45 or to the Pf 7G8 exon 1 *var* gene sequences (S1 Data) [42] using the RNA-seq aligner STAR v2.7.3a [81] allowing for a maximum mismatch of 1 (—outFilterMismatchNmax 1). Prior to read alignment, STAR was used to generate an index using the genome sequence fasta file (PlasmoDB-45_Pfalciparum7G8_Genome.fasta) or the exon 1 *var* gene sequences from S1 Data. The mapped reads in Sequence Alignment/Map (SAM) format were then summarized using the featureCounts [82] function of the Rsubread R package [83], filtering for a minimum fragment length of 85 bp for paired-end reads (minFragLength = 85), as was done previously [79]. While the annotation file PlasmoDB-45_Pfalciparum7G8.gff was used to summarize read counts across the entire Pf 7G8 genome, a simplified annotation format (SAF) file was built from *var* gene index files and supplied as annotation file to featureCounts.

The R package edgeR [84] was used to compute FPKM gene expression values using its rpkm function. The gene lengths of transcripts required for FPKM normalization were extracted from the PlasmoDB-45_Pfalciparum7G8_AnnotatedTranscripts.fasta file or the coding_nt.fa file (for the specific *var* gene assembly mapping) by indexing it using SAMtools [85] faidx and extracting the first two columns containing sequence name and length.

For strand specific visualisation the reads were aligned to the Pf 7G8 genome version 59 obtained from PlasmoDB using STAR 2.7.9a and subsequently transformed into strand specific, RPKM normalised bigwig files using deepTools 3.3.0 bamCoverage (—normalizeUsing ’RPKM’—filterRNAstrand "reverse"/"forward" -bs 50) and visualised using IGV.

Raw and normalized RNA-seq data were submitted to the BioStudies ArrayExpress collection (E-MTAB-12157).

### gDNA-seq data analysis, mapping and variant calling

The raw reads were trimmed, mapped and variant calls were made using the CLC Genomics Workbench version 21 for Linux. Since all subclonal lines were cloned just before sequencing by limiting dilution, only variant calls with >90% reads mapped to the alternative allele were considered. Manual inspection further reduced the number of reliable variants, as the AT-rich genome of Pf is prone to sequencing errors within repetitive regions. Results and raw data from gDNA-seq were submitted to the BioStudies ArrayExpress collection (E-MTAB-12158).

### Chromatin immunoprecipitation analysis (ChIP-qPCR)

Chromatin immunoprecipitation was preformed essentially as described previously [86]. Briefly, 1% paraformaldehyde-crosslinked chromatin was sheared by sonication and the soluble fraction was used for immunoprecipitation overnight using protein G coupled sepharose beads (GE Healthcare). Antibodies used for ChIP were rabbit anti-H3 (Abcam ab1791), anti-H3K9me3 (Active Motif 39161), anti-H3K27ac (Abcam ab4729), and anti-H2A.Z [24]. Washed immune complexes were eluted and de-crosslinked over night at 45°C in the presence of 500 mM NaCl. After proteinase K treatment for 1h at 37°C, the DNA was purified from each ChIP and input sample using the MinElute kit (Qiagen 28006) and analyzed by qPCR on an Applied Biosystems 7900HT fast real-time PCR system using SYBR Green PCR MasterMix (ThermoFisher Scientific 4309155) and 0.9 μM sense and antisense primers (S1 Table) in a final volume of 10 μl. ChIP enrichment at each genomic locus was calculated as % of input DNA, and histone modifications and H2A.Z were normalized relative to H3.

### Data analysis and statistics

Categorical variables were displayed as frequencies and percentages, and continuous variables as median and interquartile rage (IQR).

The differences between the two molecular methods qPCR and RNA-seq were analyzed using a Bland-Altman plot showing the agreement between two quantitative measurements. The differences between log-transformed measurements were plotted on the y-axis and the mean of the respective qPCR and RNA-seq results on the x-axis. Thus, the graph shows the deviation of the analysis results with respect to the expression level.

The expressions of *var* genes per patient were summarized in a heat map accompanied by a dendrogram of hierarchical cluster analysis applied to the expression pattern of patients. To correct for individual differences in the overall *var* transcript levels, the level for each *var* gene was normalized against total *var* transcript level in each sample. *Var* gene expressions within patient groups were summarized using the median and interquartile range (IQR). Boxplots showing the minimum, maximum, IQR, and median were used to graphically represent group expressions. Outliers, defined as values above or below the median +/- 1.5 times the IQR, are plotted outside the whiskers of the boxplot. Correlations between individual *var* gene expressions are calculated using the Pearson correlation coefficient (PCC) with the log-transformed measurements.

## Supporting information

S1 Fig Schematics of the MAVACHE and CVac-Tü3 study designs. (PDF)

S2 Fig Ranking of *var* genes according to transcript levels detected in samples from malaria-naïve volunteers infected with PfSPZ 7G8. The median *var* transcript level relative to the *arginyl-tRNA synthetase* transcript level with IQR is shown for 11 volunteer samples. Group affiliation of *var* genes is indicated by the color code: A-type *var* genes in red, the subfamily *var1* in dark red, B-type genes in blue, group C genes in green, and the *var2csa* gene (group E) in yellow.
(PDF)

S3 Fig Strand-specific RNA-seq data. (**A**) Heat map showing strand-specific expression data from all 7G8 *var* gene loci in Sanaria cell bank ring stage parasites (aliquot B, 8. Generation). Strand-specific mapping of RNA-seq reads to the 7G8 reference genome (version 59). Scale bar indicates strand-specific bam file read coverage over 50 bp bins normalized to RPKM. Group affiliation of *var* genes is indicated by the color code: A-type *var* genes in red, the subfamily *var1* in dark red, B-type genes in blue, group C genes in green, and the *var2csa* gene (group E) in yellow. On PlasmoDB annotated pseudogenes are marked with asterisk. The orientation of each gene is indicated in brackets after the accession number. (**B**) Forward and reverse strand profiles of transcribed *var* genes in cell bank A parasites (IGV). Scales were adjusted to depict low level antisense lncRNA transcripts.
(PDF)

S4 Fig *Var* gene expression profiles of 7G8 cell bank parasites derived from Sanaria (aliquot A and B) and BEI Resources (MRA-152, MRA-926, MRA-154). (A) Heat map showing *var* expression of 7G8 parasites from Sanaria cell bank aliquots A and B and of three 7G8 aliquots deposited at BEI resources by different providers. Expression of each *var* gene is normalized to expression of *arginyl-tRNA synthetase*. (B) Pie charts showing the proportion of *var* gene expression by group for the different 7G8 lines. The names of *var* genes are indicated, and *var* gene groups are colored according to the scheme: A in red, A-*var1* in dark red, B in blue, C in green and E (*var2csa*) in yellow.
(PDF)

S5 Fig *Var* gene expression profiles of CSA-selected 7G8 cell bank parasites after cultivation for up to 100 replication cycles after selection. Heat map showing the *var* expression of 7G8 cell bank parasites (aliquot A) selected on CSA to express *var2csa* after cultivation for up to 100 parasite replications. The expression of each *var* gene is normalized against the expression of *arginyl-tRNA synthetase*. The names of *var* genes are indicated, and *var* gene groups are colored according to the scheme: A in red, A-*var1* in dark red, B in blue, C in green and E (*var2csa*) in yellow.
(PDF)

S6 Fig qPCR on gDNA from 7G8 subclones to confirm presence of each *var* gene variant in the different genomes. Shown are raw Ct values from about 2.5 ng gDNA used as template per qPCR reaction. The Ct values from the cell bank aliquot A bulk culture are marked in black for reference, and a line is drawn at the mean.
(PDF)

S7 Fig Comparison of *var* gene expression in ring and gametocytes stages determined by qPCR. (**A**) Representative Giemsa smears of gametocyte cultures from Sanaria cell bank aliquot A +/- enriched for CSA binding (’*Sanaria A*’, ’*Sanaria A CSA*’) and subclonal lines A2E10 and A1G9 used for ChIP and *var*-qPCR. (**B**) Comparison of *var* gene expression determined by qPCR between ring and gametocyte stages using RNA collected in parallel to ChIP experiments (data shown in Fig 4) for Sanaria cell bank aliquot A enriched for CSA binding (’*Sanaria A CSA*’) and subclones A2E10 and A1G9. The *var* genes are sorted by group in ascending order. *Var* gene groups are colored according to the scheme: A in red, A-*var1* in dark, red, B in blue, C in green and E (*var2csa*) in yellow.
(PDF)

S1 Table The 7G8 *var* gene repertoire and the domain composition of the encoding PfEMP1 variants according to the classification from Rask *et al*. [5]. Question marks indicate unknown features of the *var* gene/PfEMP1. Gene IDs and gene type annotation according to PlasmoDB Release 58. ATS: Acidic terminal segment; CIDR: Cysteine-rich interdomain region; DBL: Duffy-binding like domain; NTS: N-terminal segment; UPS: upstream sequence.
(XLSX)

S2 Table Oligonucleotides used in this study. (XLSX)

S3 Table qPCR data on 7G8 *var* gene expression in pre-mosquito cell bank parasites (A) and in parasites from malaria-naïve volunteers (B).
(XLSX)

S4 Table *Var* gene expression in MRA-152, 7G8 pre-mosquito cell bank parasites (aliquot A) and 7G8 subclones before and after CSA selection. (XLSX)

S5 Table Unique genomic variants of 7G8 subclones A1G9 (*var2csa*-expressing subclone, control) versus A2G2 and A2E10 (Pf7G8_040025600-expressing subclones) determined by gDNA-seq. (XLSX)

S6 Table ChIP-qPCR and *var* expression profiles from 7G8 pre-mosquito cell bank parasites (aliquot A) and 7G8 subclones before and after CSA selection. (XLSX)

S1 Data All *var* exon 1 sequences used for design of the oligonucleotides and RNA-seq mapping (coding_nt.fa) [42]. (FA)
