# Supplementary material for: The exception that proves the rule: Virulence gene expression at the onset of Plasmodium falciparum blood stage infections
Source: PLoS Pathog. 2023 Jun 29;19(6):e1011468. doi: 10.1371/journal.ppat.1011468 (PMC10337978; doi:10.1371/journal.ppat.1011468)
Supplement: S1 Fig — (PDF) [file ppat.1011468.s001.pdf]

Figure S1

MAVACHE

Sequential optimization of dose and schedule of PfSPZ Vaccine, verified by randomized, controlled, double-blind immunization and controlled human malaria infection in malaria-naïve, healthy adult volunteers in Germany

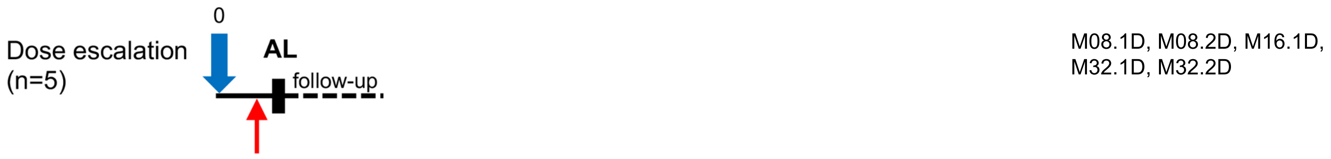

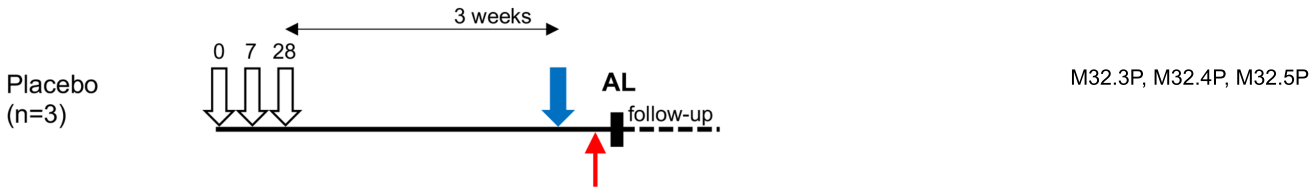

CVac-Tü3

Safety and protective efficacy of a simplified *P. falciparum* sporozoite Chemoprophylaxis Vaccine (PfSPZ-CVac) regimen in healthy malaria-naïve adults in Germany

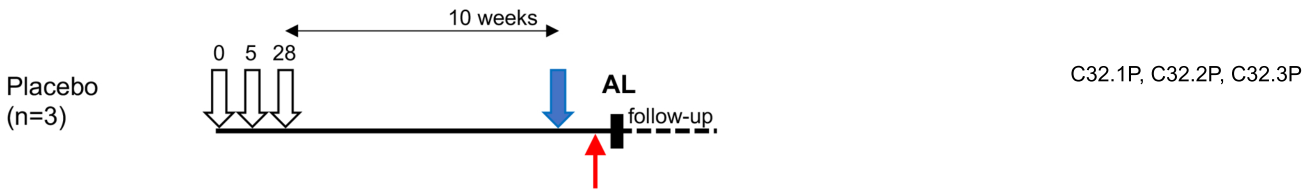

Placebo immunization

Challenge with 3,200 PfSPZ 7G8

Sampling

Artemether/lumefantrine (AL) treatment after three consecutive parasitemia measurements with at least one >100 parasites/mL (qPCR), or on day 21
