# Supplementary figures and images for: The exception that proves the rule: Virulence gene expression at the onset of Plasmodium falciparum blood stage infections
Source: PLoS Pathog. 2023 Jun 29;19(6):e1011468. doi: 10.1371/journal.ppat.1011468 (PMC10337978; doi:10.1371/journal.ppat.1011468)

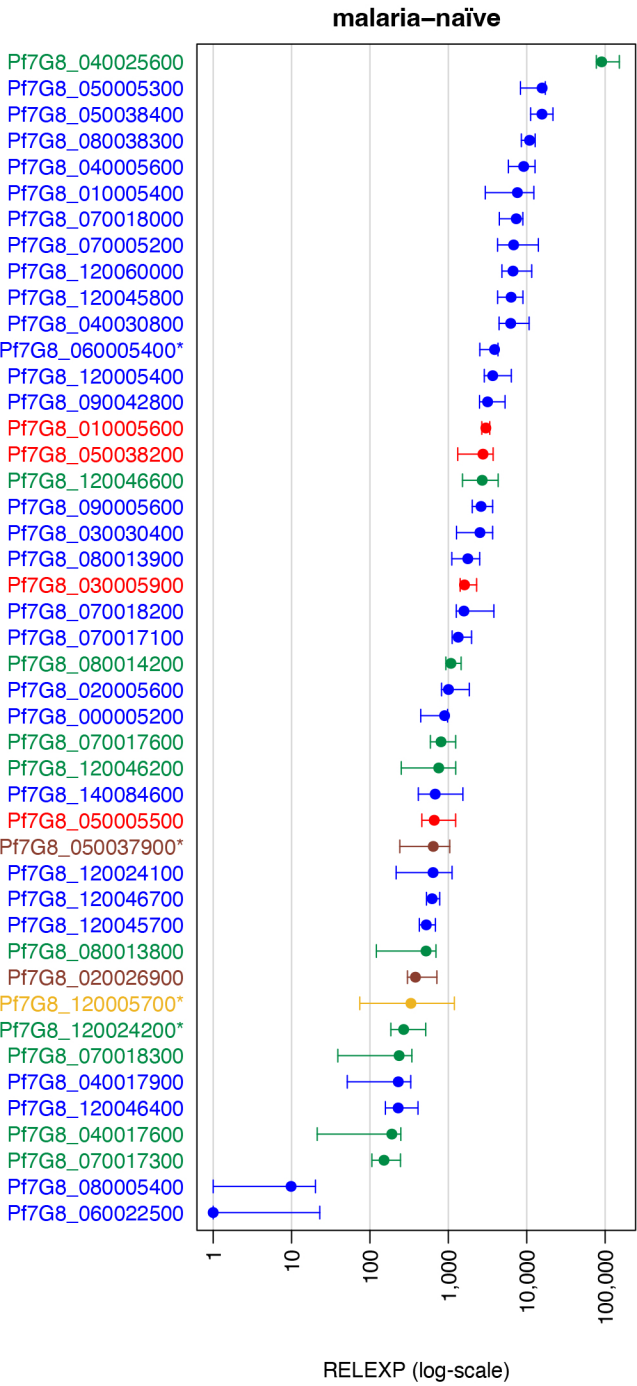

Supplement: S2 Fig — The median var transcript level relative to the arginyl-tRNA synthetase transcript level with IQR is shown for 11 volunteer samples. Group affiliation of var genes is indicated by the color code: A-type var genes in red, the subfamily var1 in dark red, B-type genes in blue, group C genes in green, and the var2csa gene (group E) in yellow. (PDF) [file ppat.1011468.s002.pdf]

A

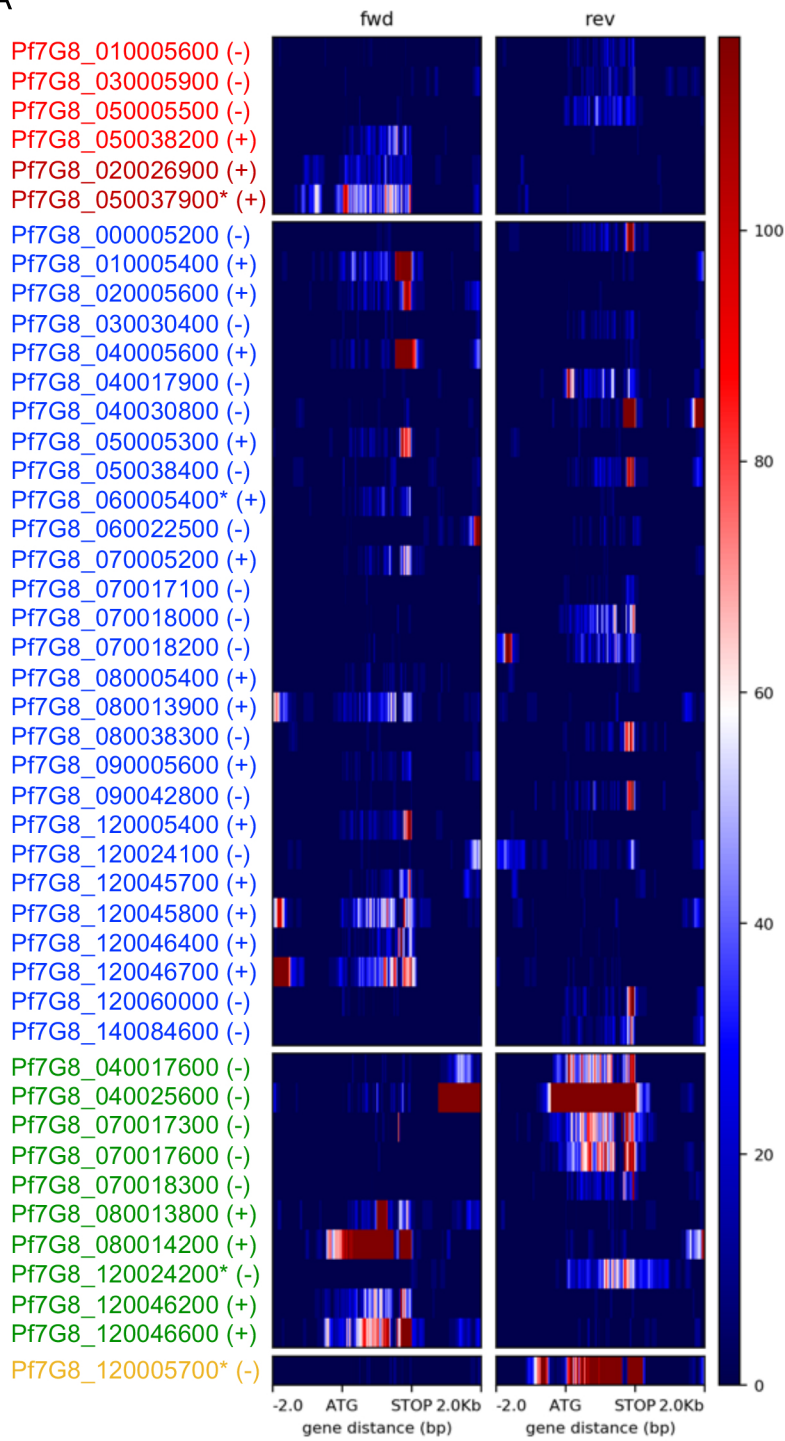

B

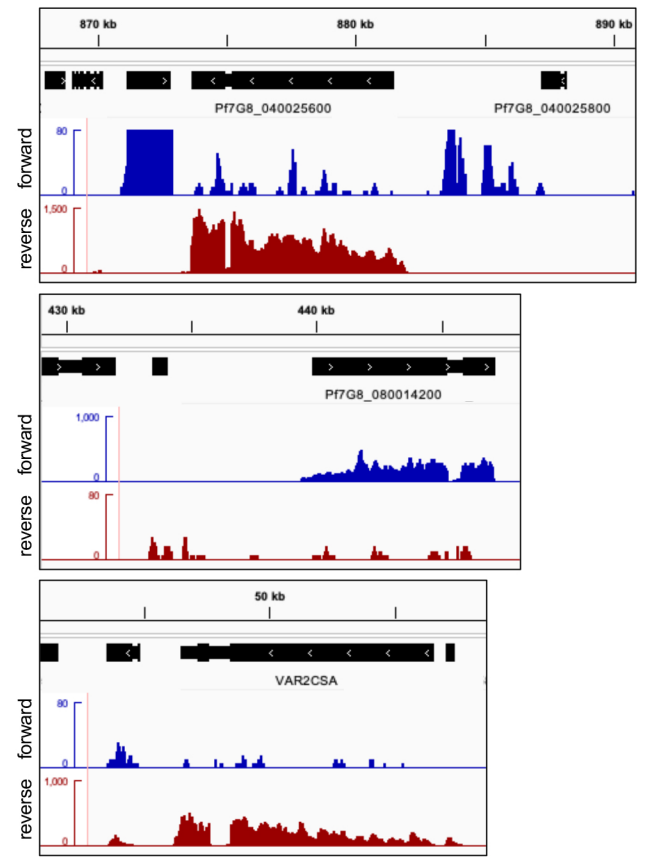

Supplement: S3 Fig — (A) Heat map showing strand-specific expression data from all 7G8 var gene loci in Sanaria cell bank ring stage parasites (aliquot B, 8. Generation). Strand-specific mapping of RNA-seq reads to the 7G8 reference genome (version 59). Scale bar indicates strand-specific bam file read coverage over 50 bp bins normalized to RPKM. Group affiliation of var genes is indicated by the color code: A-type var genes in red, the subfamily var1 in dark red, B-type genes in blue, group C genes in green, and the var2csa gene (group E) in yellow. On PlasmoDB annotated pseudogenes are marked with asterisk. The orientation of each gene is indicated in brackets after the accession number. (B) Forward and reverse strand profiles of transcribed var genes in cell bank A parasites (IGV). Scales were adjusted to depict low level antisense lncRNA transcripts. (PDF) [file ppat.1011468.s003.pdf]

Figure S4

A

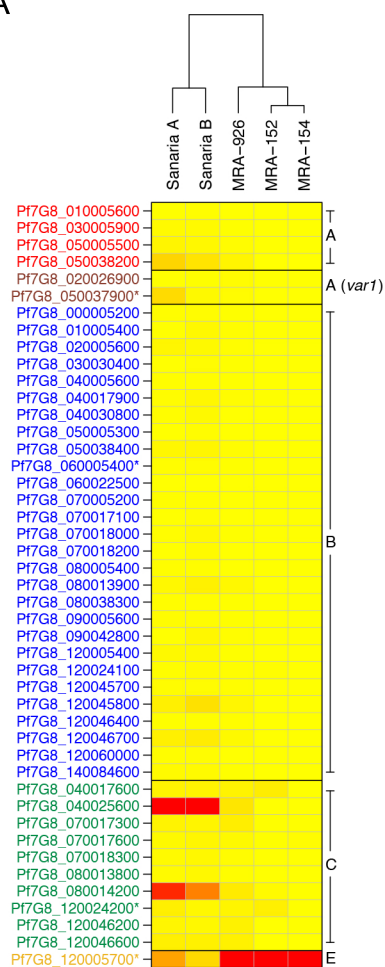

B

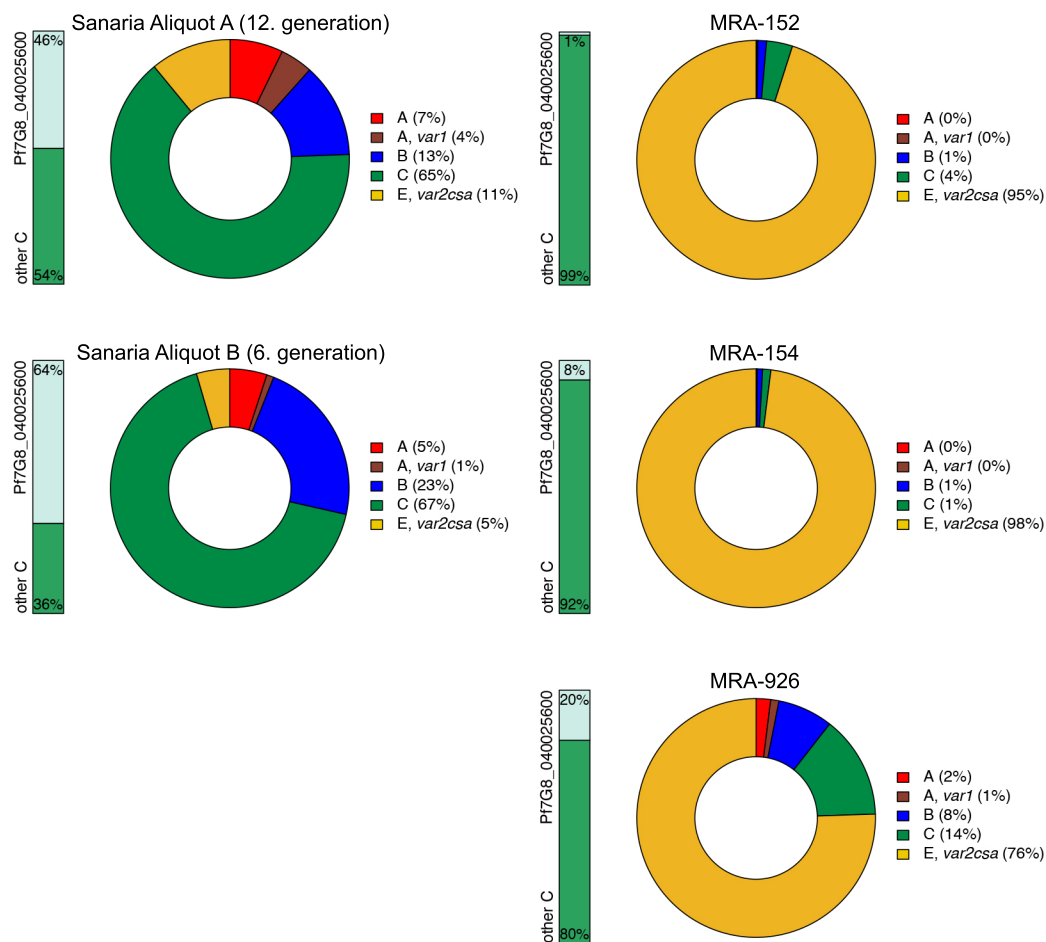

Supplement: S4 Fig — (A) Heat map showing var expression of 7G8 parasites from Sanaria cell bank aliquots A and B and of three 7G8 aliquots deposited at BEI resources by different providers. Expression of each var gene is normalized to expression of arginyl-tRNA synthetase. (B) Pie charts showing the proportion of var gene expression by group for the different 7G8 lines. The names of var genes are indicated, and var gene groups are colored according to the scheme: A in red, A-var1 in dark red, B in blue, C in green and E (var2csa) in yellow. (PDF) [file ppat.1011468.s004.pdf]

Figure S5

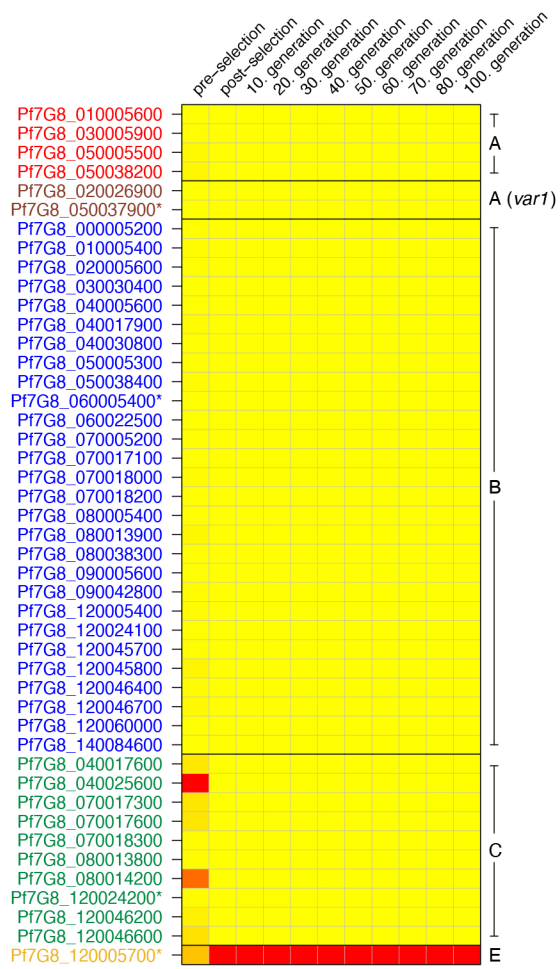

Supplement: S5 Fig — Heat map showing the var expression of 7G8 cell bank parasites (aliquot A) selected on CSA to express var2csa after cultivation for up to 100 parasite replications. The expression of each var gene is normalized against the expression of arginyl-tRNA synthetase. The names of var genes are indicated, and var gene groups are colored according to the scheme: A in red, A-var1 in dark red, B in blue, C in green and E (var2csa) in yellow. (PDF) [file ppat.1011468.s005.pdf]

Figure S6

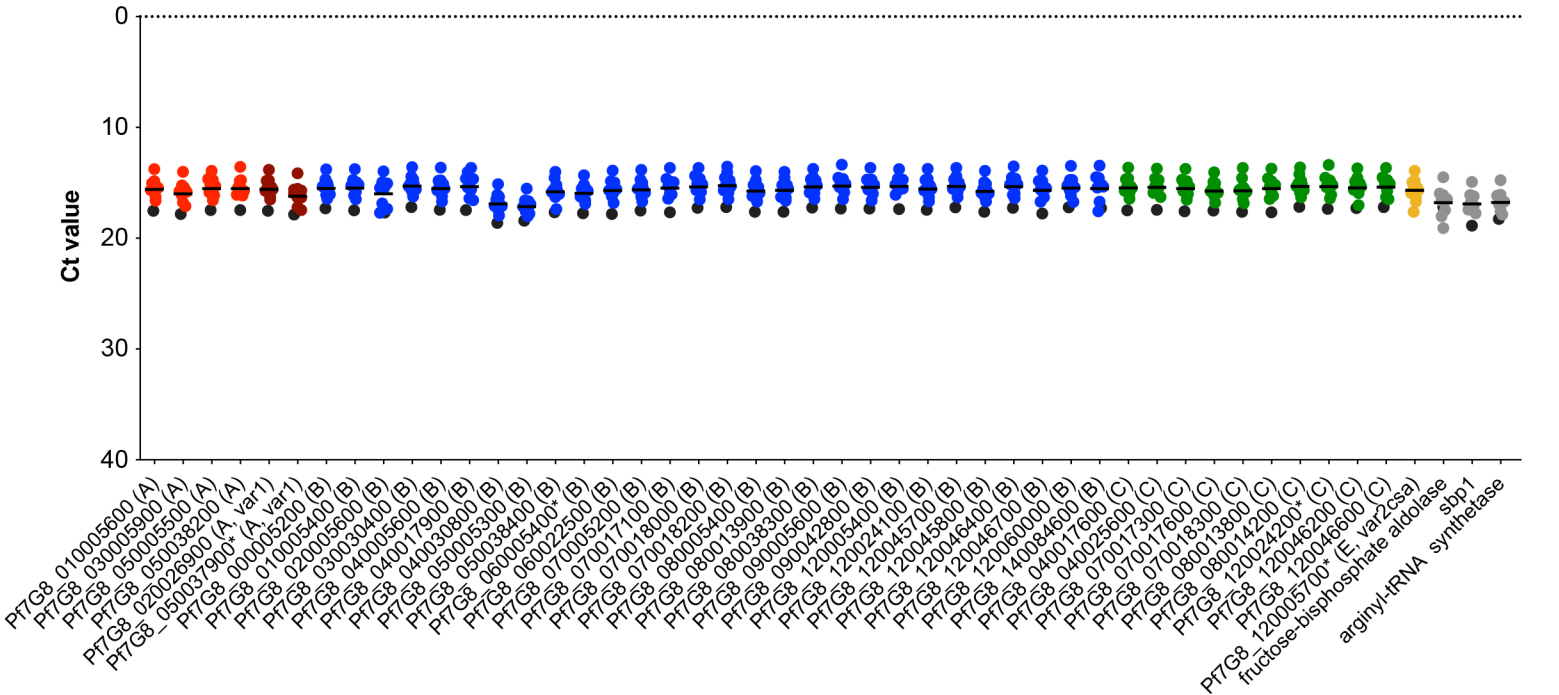

Supplement: S6 Fig — Shown are raw Ct values from about 2.5 ng gDNA used as template per qPCR reaction. The Ct values from the cell bank aliquot A bulk culture are marked in black for reference, and a line is drawn at the mean. (PDF) [file ppat.1011468.s006.pdf]

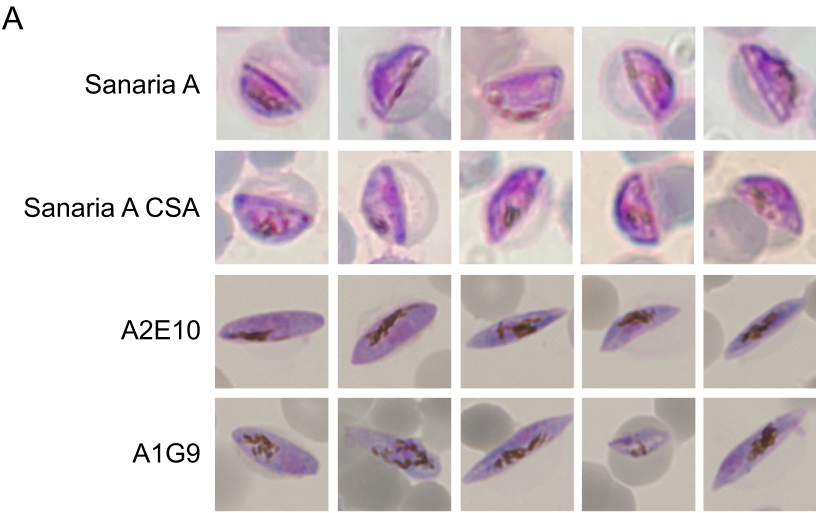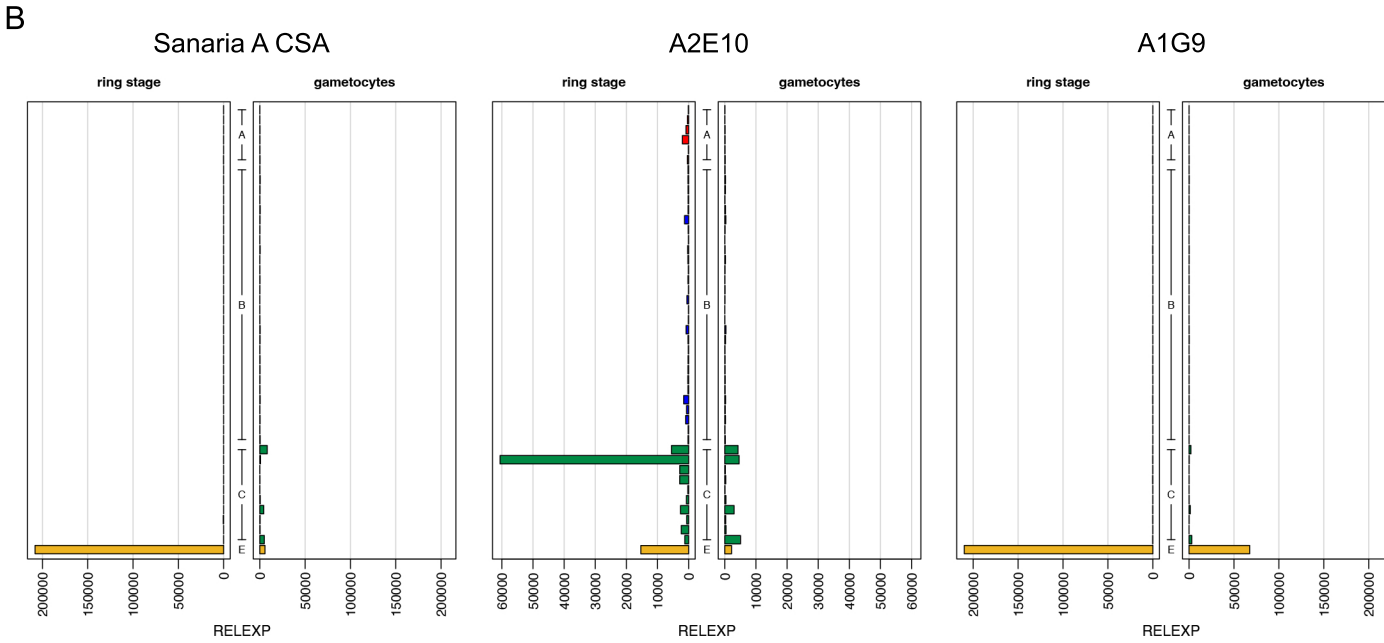

Supplement: S7 Fig — (A) Representative Giemsa smears of gametocyte cultures from Sanaria cell bank aliquot A +/- enriched for CSA binding (’Sanaria A’, ’Sanaria A CSA’) and subclonal lines A2E10 and A1G9 used for ChIP and var-qPCR. (B) Comparison of var gene expression determined by qPCR between ring and gametocyte stages using RNA collected in parallel to ChIP experiments (data shown in Fig 4) for Sanaria cell bank aliquot A enriched for CSA binding (’Sanaria A CSA’) and subclones A2E10 and A1G9. The var genes are sorted by group in ascending order. Var gene groups are colored according to the scheme: A in red, A-var1 in dark, red, B in blue, C in green and E (var2csa) in yellow. (PDF) [file ppat.1011468.s007.pdf]
